# Supplementary material for: Spatial Transcriptomics to define transcriptional patterns of zonation and structural components in the mouse liver
Source: Nat Commun. 2021 Dec 2;12:7046. doi: 10.1038/s41467-021-27354-w (PMC8640072; doi:10.1038/s41467-021-27354-w)
Supplement: Supplementary file 1 — Supplementary Information [file 41467_2021_27354_MOESM1_ESM.pdf]

Supplementary figure 1: Quantification of cell type proportions by immunofluorescence assay (IFA). a depicts a representative fluorescence image of a spot used for manual counting of Kupffer cells and hepatocytes of a total of 224 independent imaging and counting experiments. The white circle shares the dimensions of a spot on a ST array. DNA was stained using Hoechst (left). Kupffer cells were counted based on a positive signal for DNA and F4/80 signal (middle, left in green). Hepatocytes were counted based on a positive signal for HNF4α and Hoechst signal (middle, right, red). The merged image to the right depicts the overlay of all three signals within the region of interest. White scale bars indicate 10 µm. **b** Similar to **a**, depicts a representative fluorescence image of a spot used for manual counting of Kupffer cells and endothelial cells in the region of interest (white circle) of a total of 224 independent imaging and counting experiments. DNA is represented in blue to the left, Kupffer cells are represented in green (middle, left). Endothelial cells were counted based on a positive signal for CD31 and Hoechst signal (middle, right, red). The merged image to the right depicts the overlay of all three signals within the region of interest. White scale bars indicate 10 µm. **c** Shows the distribution of the manual counting of 100 µm spots for nuclear Hoechst staining. 250 spots were randomly selected, 26 spots were excluded due to tissue damage or spots assigned outside of the tissue area, resulting in 224 counted spots. **d** HNF4α/Hoechst-positive hepatocytes were counted in 110 spots, **e** Kupffer cells were counted in 224 spots. **f** Endothelial cells were counted in 114 spots. Source data are provided as Source Data file.

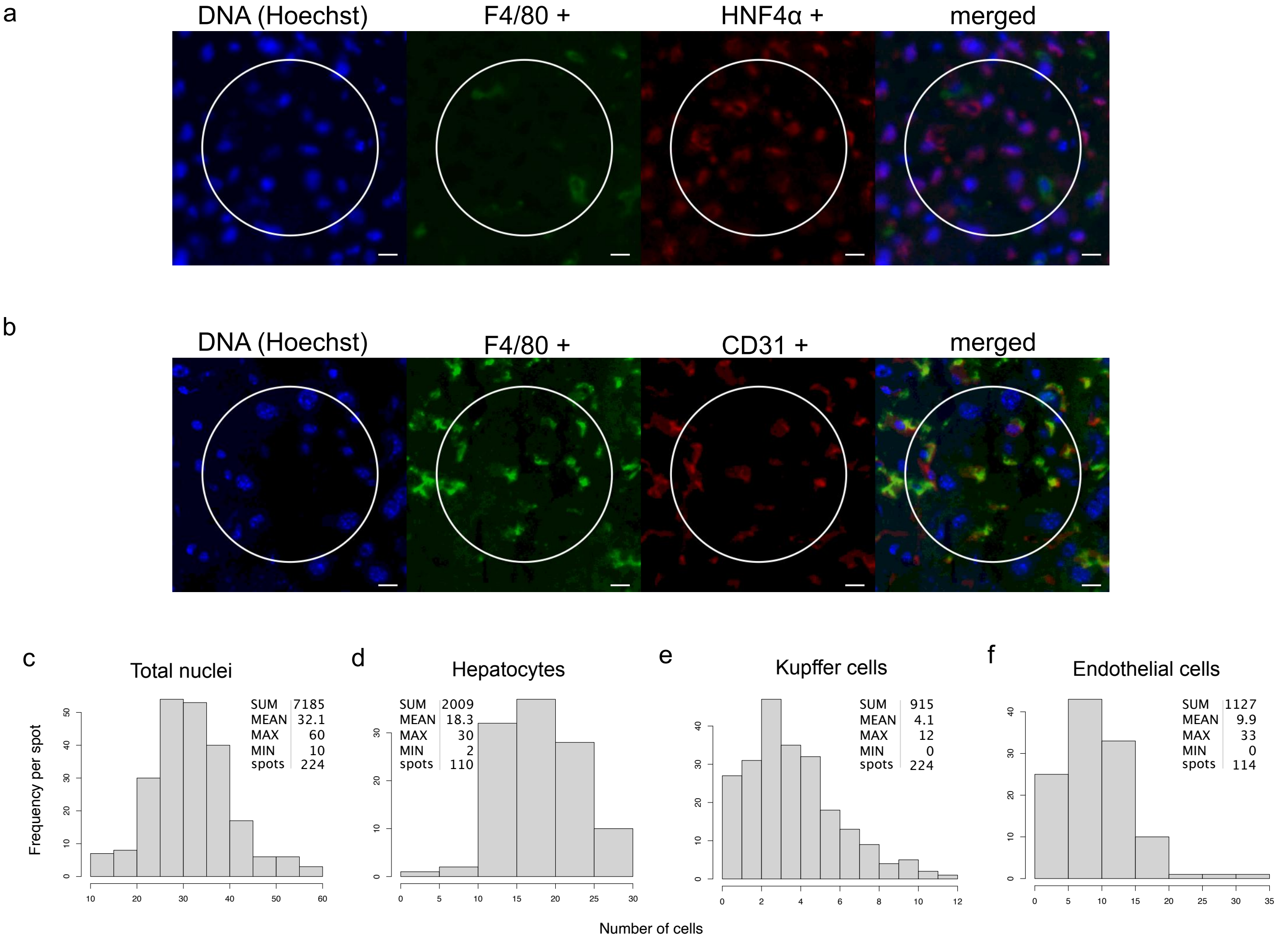

Supplementary figure 2: Distribution of the number of unique transcripts across clusters. **a** The logarithmic number of unique transcripts (base10) for each cluster reveals equal numbers of unique transcripts for each cluster. **b** The log10 of unique transcripts exhibits a uniform distribution in the UMAP embedding of spatial data and across cluster annotations. Transcript numbers are ranging from low (light) to high (dark) values.

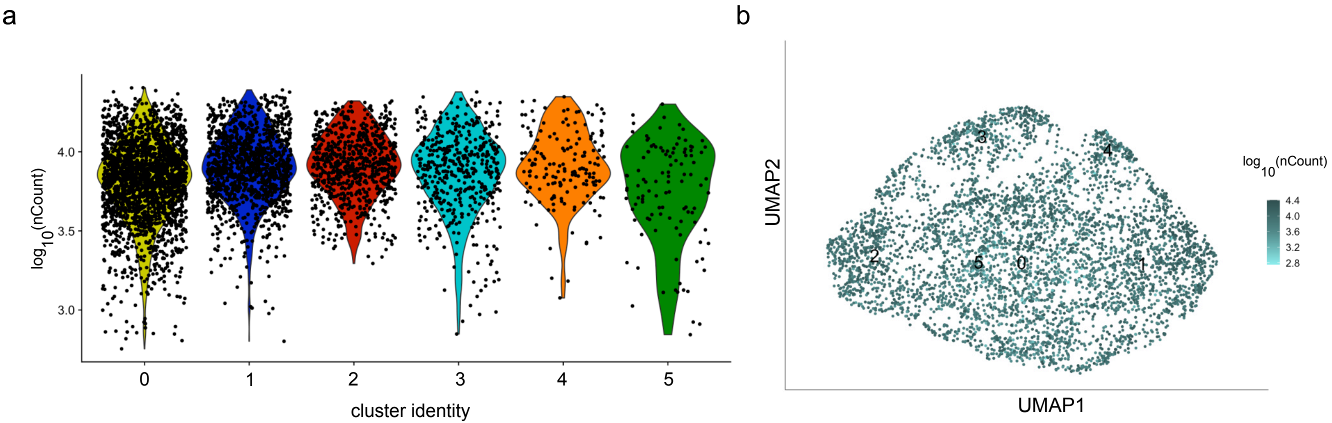

Supplementary figure 3: Visualization of unsupervised clustering results of integrated sequencing data on spots under the tissue for **a** sample 1 of three sections of a part of a caudate lobe **b** sample 2 of three sections of a part of a caudate lobe and **c** sample 3 of two sections of a part of a right lobe.

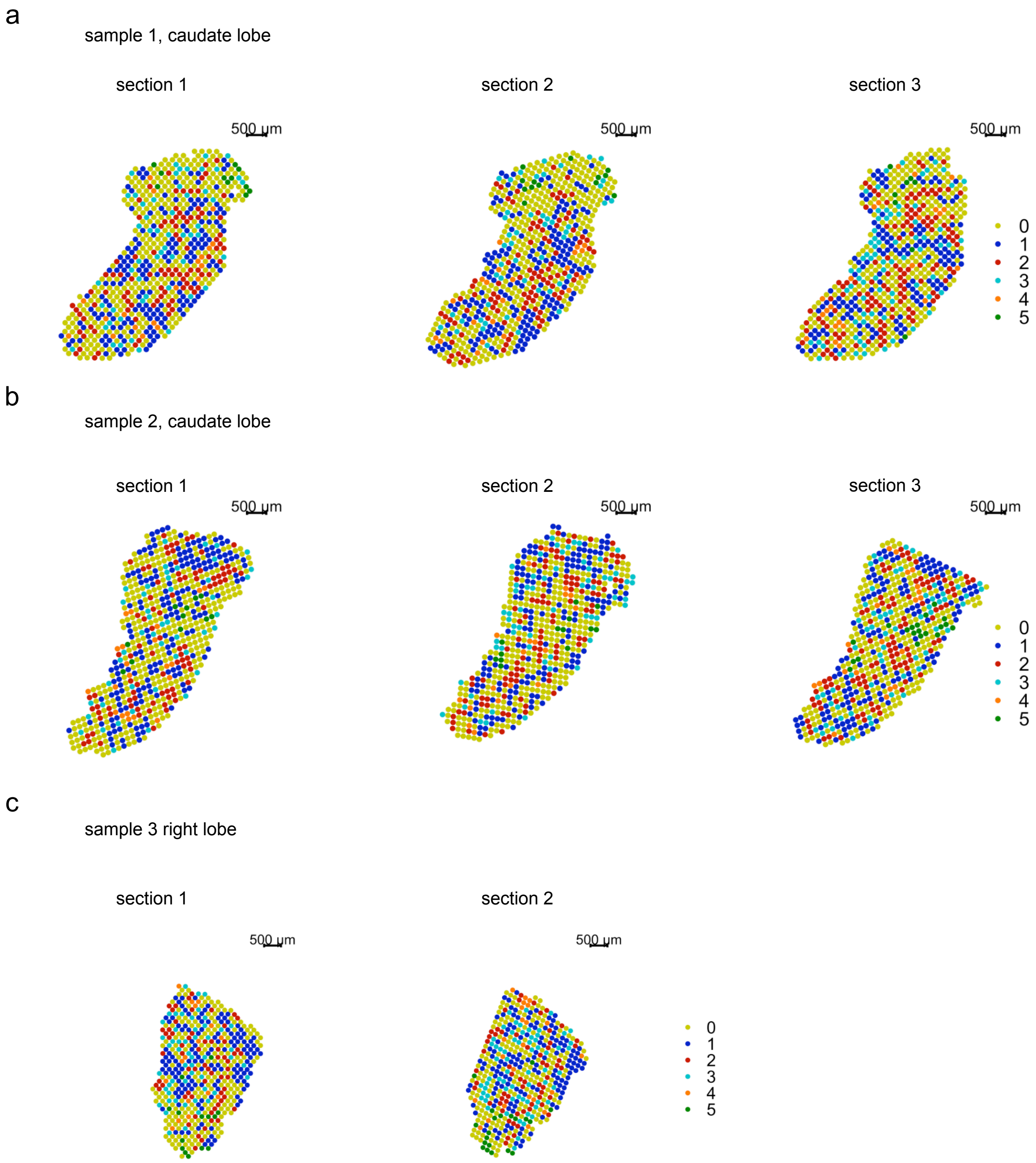

Supplementary figure 4: For spots belonging to each cluster identity (0-5) the most frequent cluster identities of the four spots within the closest proximity (1500 µm) were determined, ranging from fractions between 0 and 0.5 of all neighboring spots of each cluster. The cluster for which the neighboring fractions are determined is depicted in grey with a red dashed stroke. Dashed lines in magenta indicate the expected distribution of fractions among the neighboring clusters if the spots under the tissue were randomly assorted.

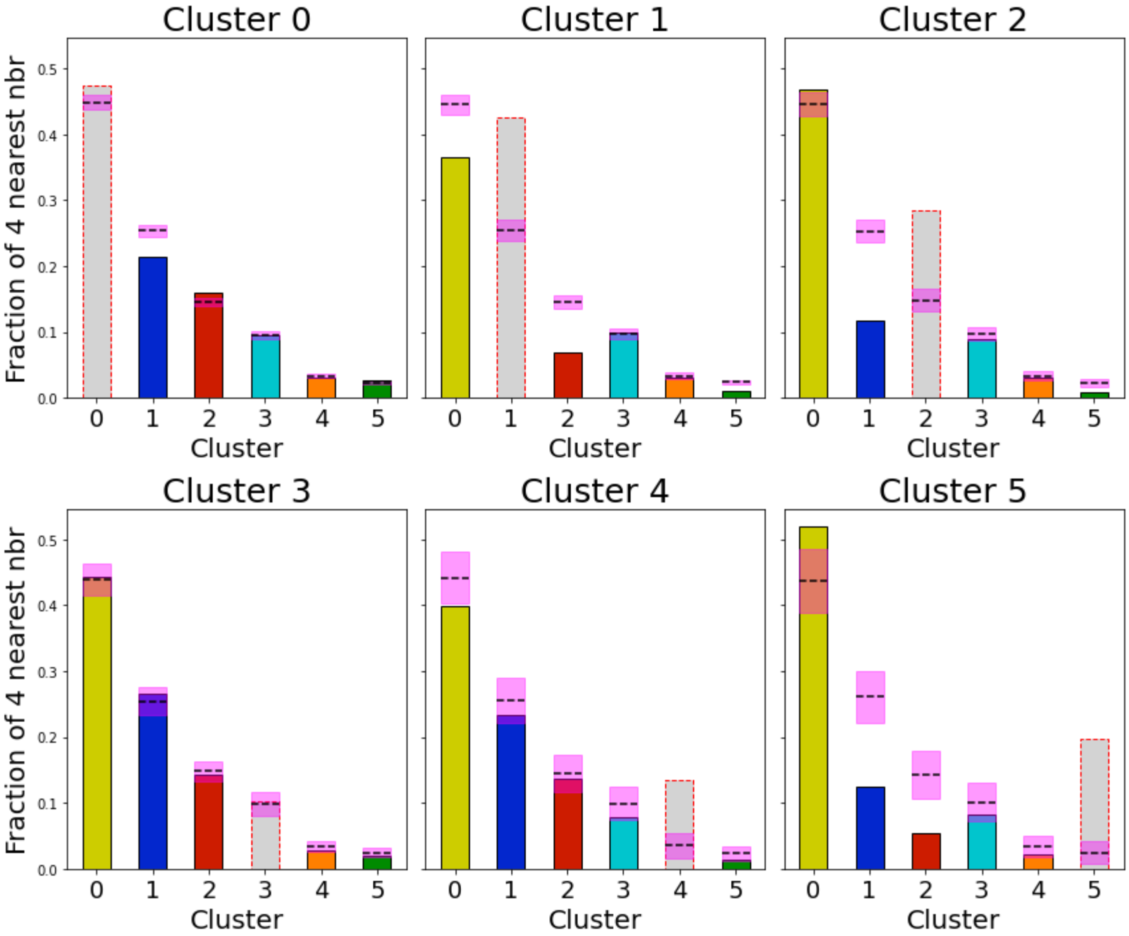

Supplementary figure 5: Visualization of single cell data integration by *stereoscope* on spots under the tissue for all sections of this study. Spot-wise calculated proportion-values were scaled using quantile scaling between values of 0 to 1 for all 20 annotated cell types of the MCA single-cell data and each section. Proportion-values are visualized on spots under the tissue for section 1 (top) and section 2 (bottom) of the right lobe (sample 3).

sample 3, right lobe

section 1

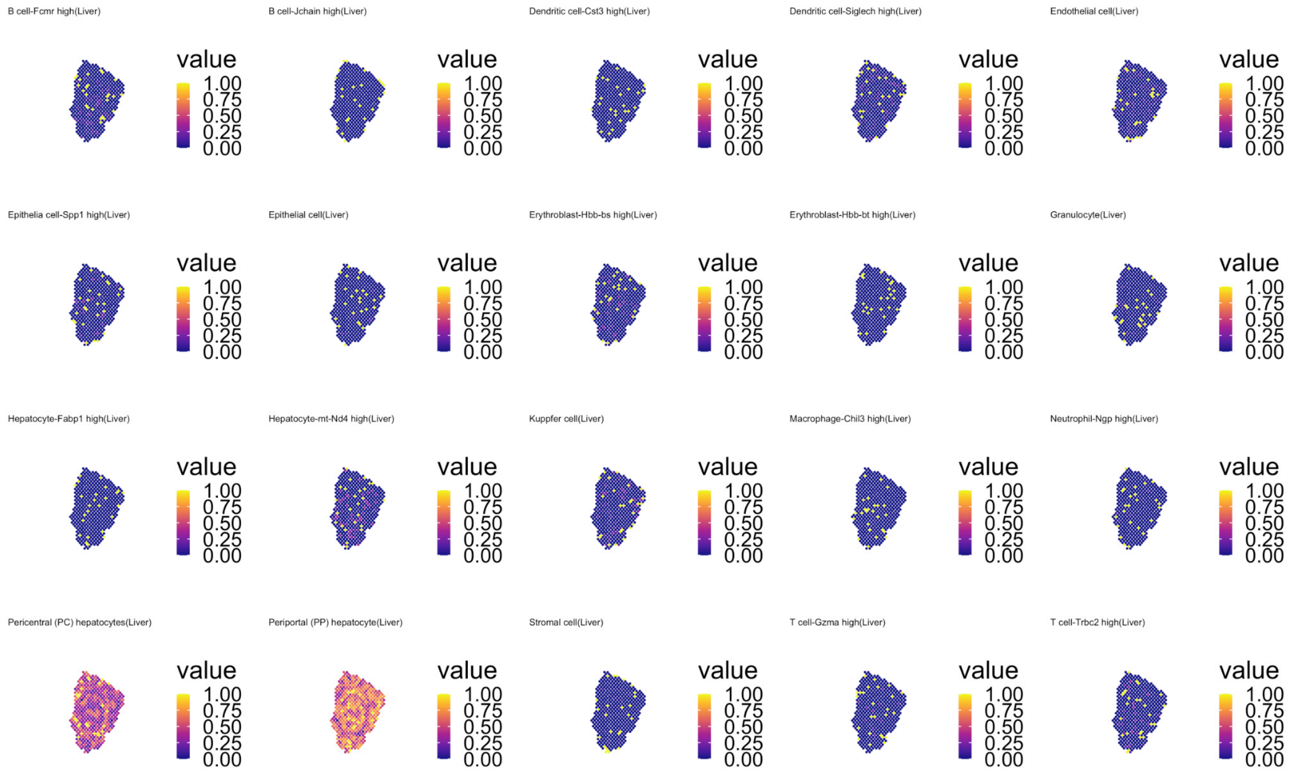

section 2

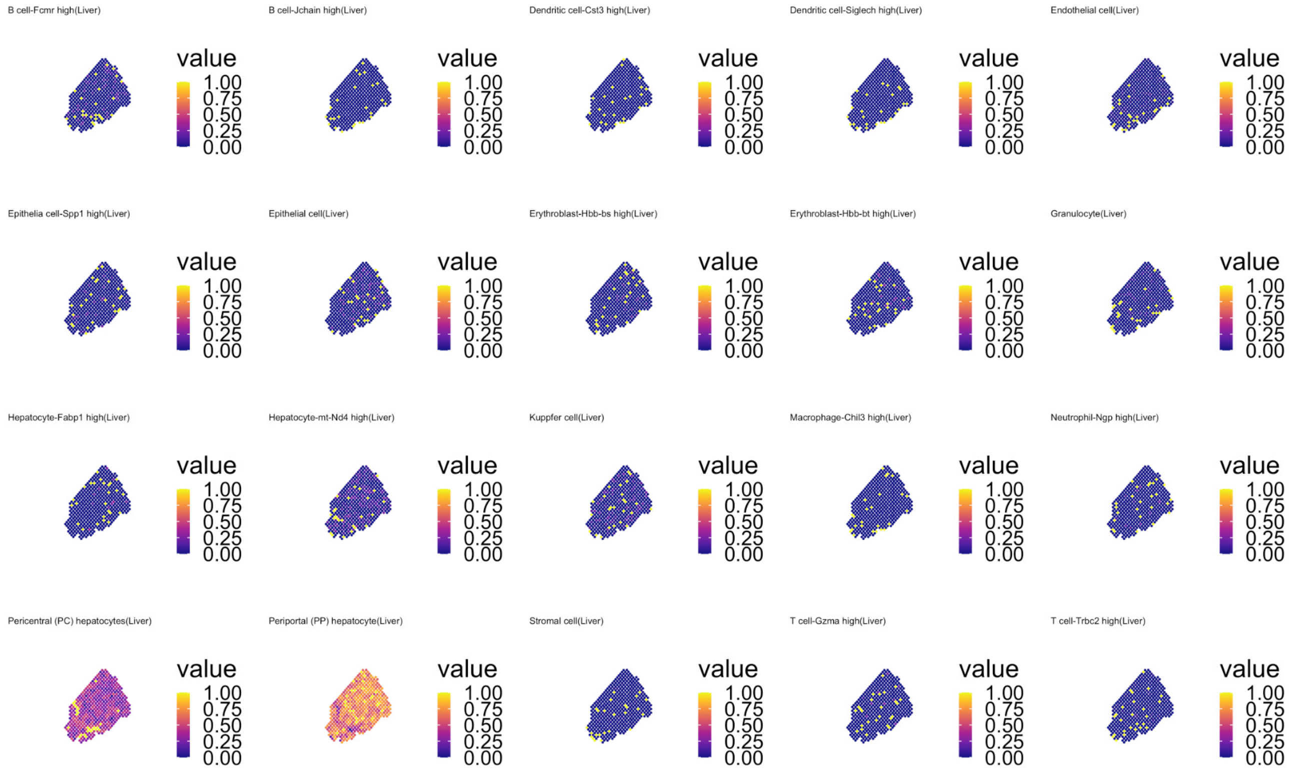

Supplementary figure 6: Visualization of single cell data integration by *stereoscope* on spots under the tissue for all sections of this study. Spot-wise calculated proportion-values were scaled using quantile scaling between values of 0 to 1 for all 20 annotated cell types of the MCA single-cell data and each section. Proportion-values are visualized on spots under the tissue for Proportion-values are visualized on spots under the tissue for section 1 (top) to section 3 (bottom) of the caudate lobe (sample 1).

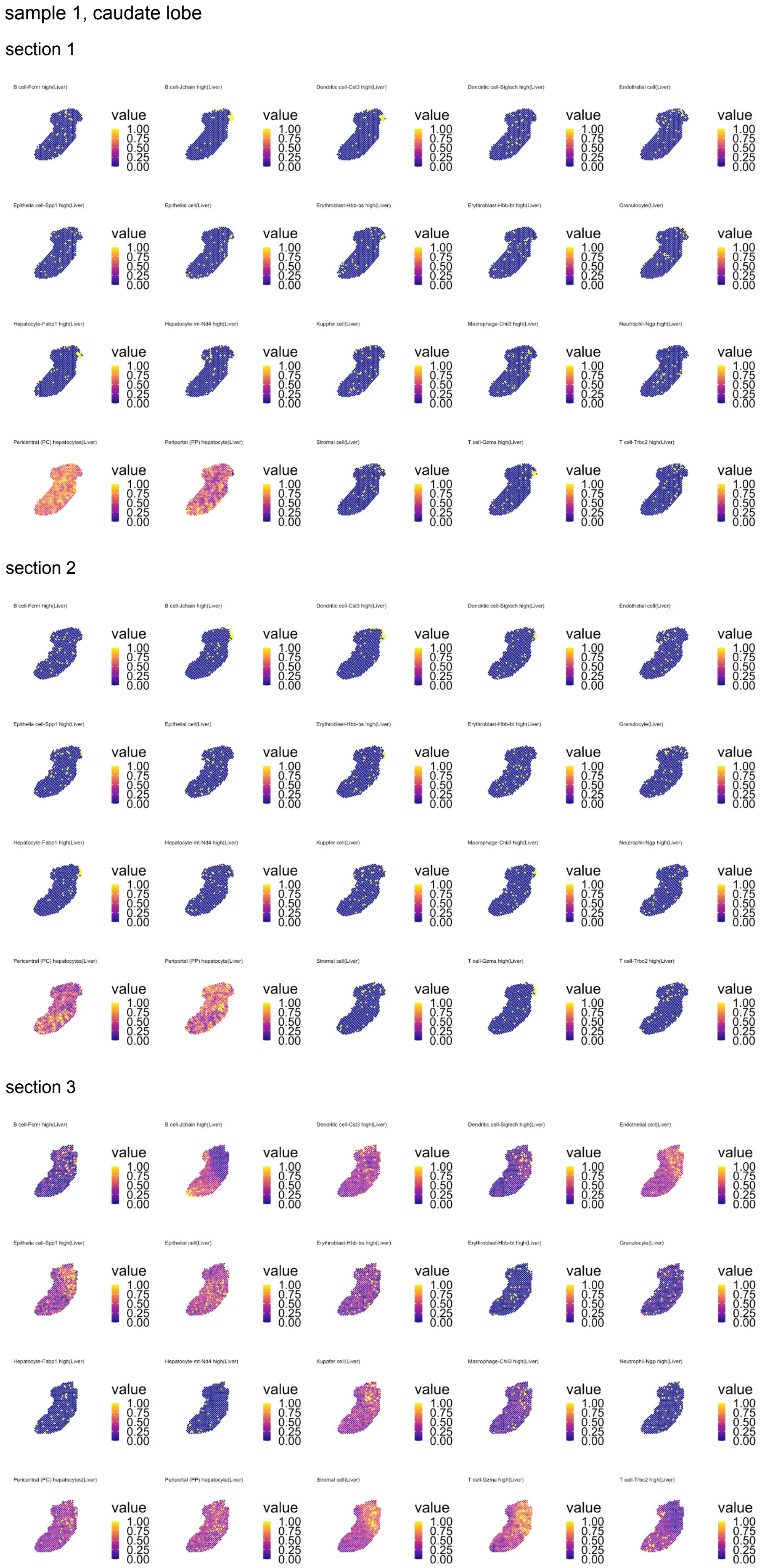

Supplementary figure 7: Visualization of single cell data integration by *stereoscope* on spots under the tissue for all sections of this study. Spot-wise calculated proportion-values were scaled using quantile scaling between values of 0 to 1 for all 20 annotated cell types of the MCA single-cell data and each section. Proportion-values are visualized on spots under the tissue for Proportion-values are visualized on spots under the tissue for section 1 (top) to section 3 (bottom) of the caudate lobe (sample 2).

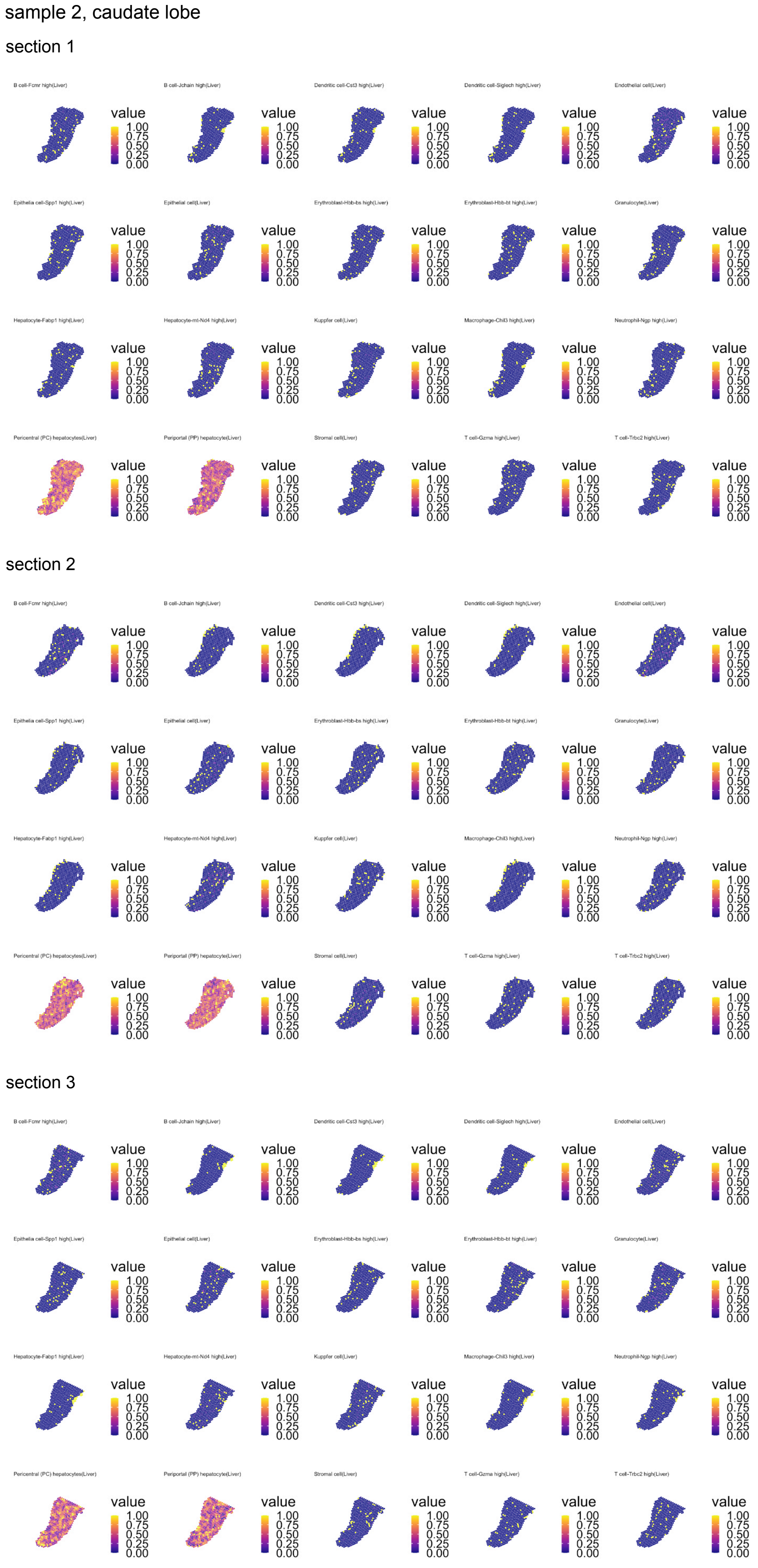

Supplementary figure 8: Spearman correlations between single cell periportal and pericentral cell-types and pericentral and periportally annotated spots in spatial transcriptomics data. **a** Spearman correlations between module score values of markers from spatial transcriptomics data (cluster 1 periportal scores, cluster 2 pericentral scores) and module score values of marker genes from single cell (periportal hepatocytes scores, pericentral hepatocyte scores) using spatial transcriptomics count-data. **b** Spearman correlations between module score values of markers from spatial transcriptomics data (cluster 1 periportal scores, cluster 2 pericentral scores) and module score values of marker genes from single cell (periportal hepatocytes scores, pericentral hepatocyte scores) using mouse cell atlas (MCA) data [39].

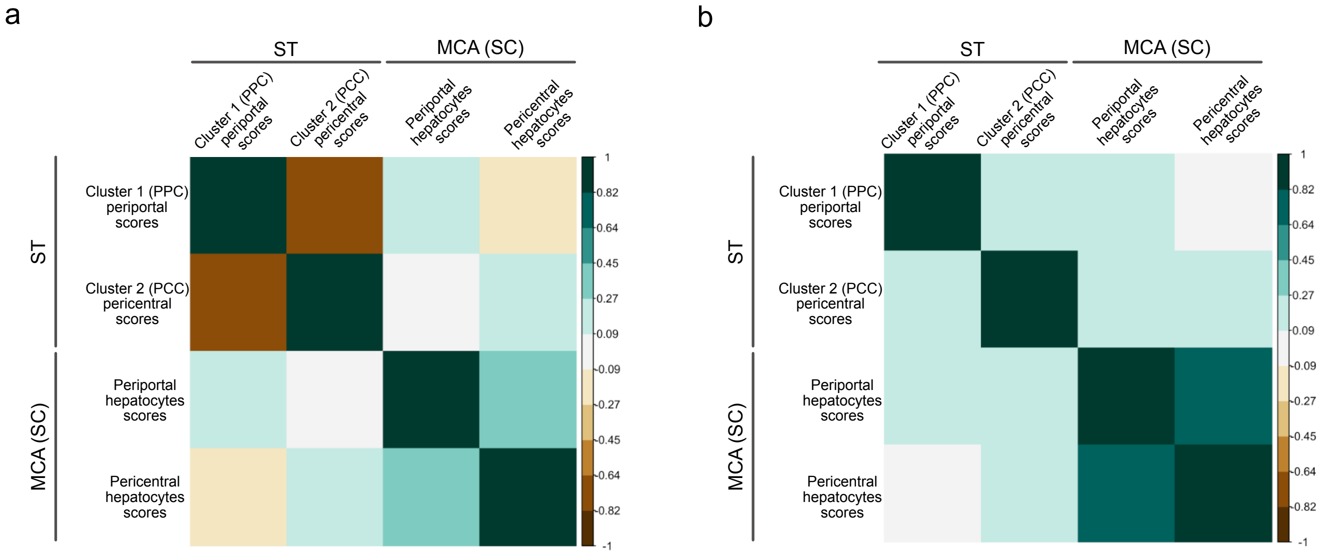

Supplementary figure 9: Pearson correlation of marker genes of all clusters ordered by the first principal component (corrplot package (v.0.84), see methods).

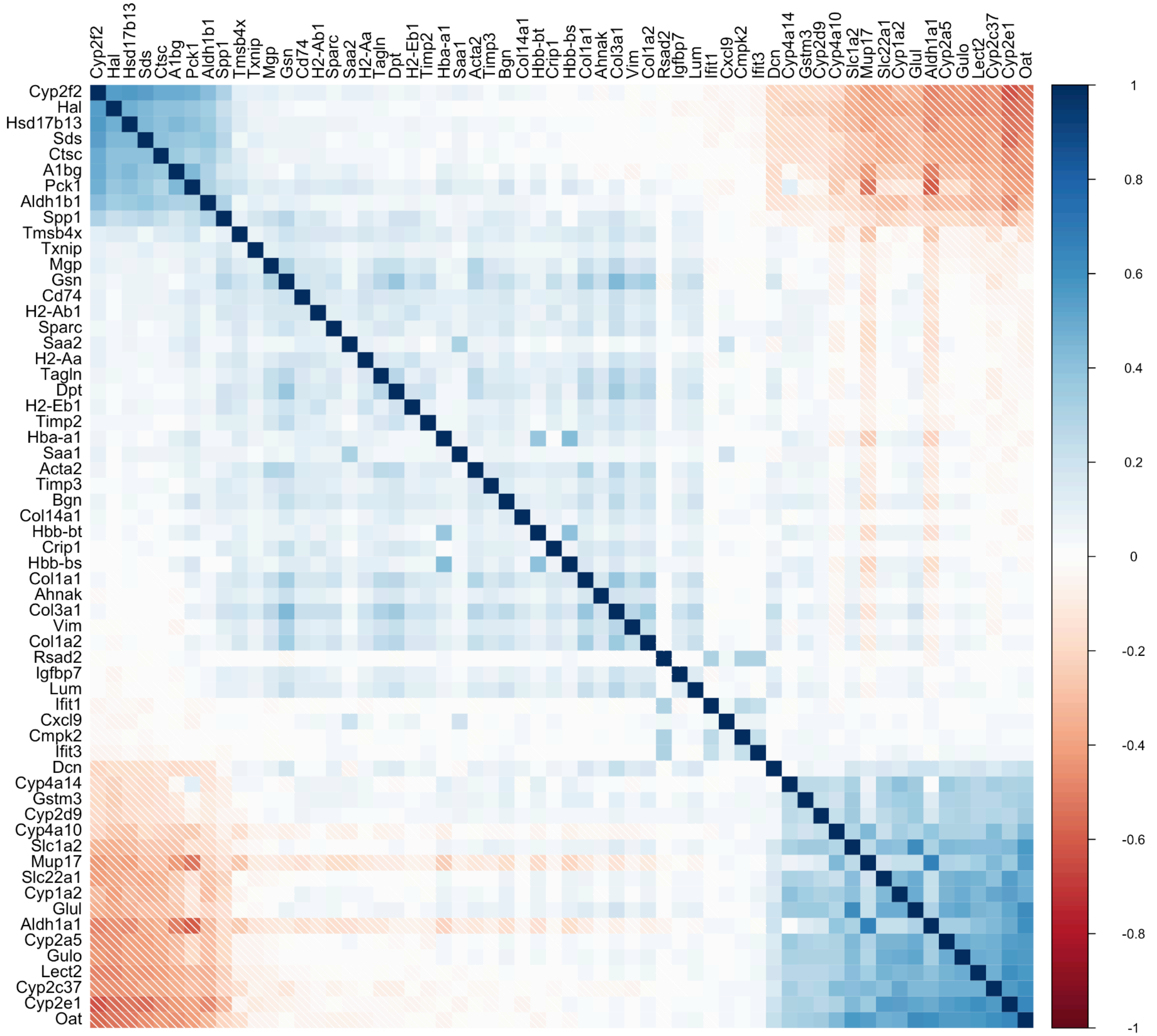

Supplementary figure 10: Spatial autocorrelation of marker genes. Bar plot of genes with spatial autocorrelation above a threshold of 0.2 for spatial autocorrelation. Higher values indicate stronger spatial correlation of the gene, while lower values indicate a more random distribution of the gene across the tissue. The color refers to the identified cluster identity.

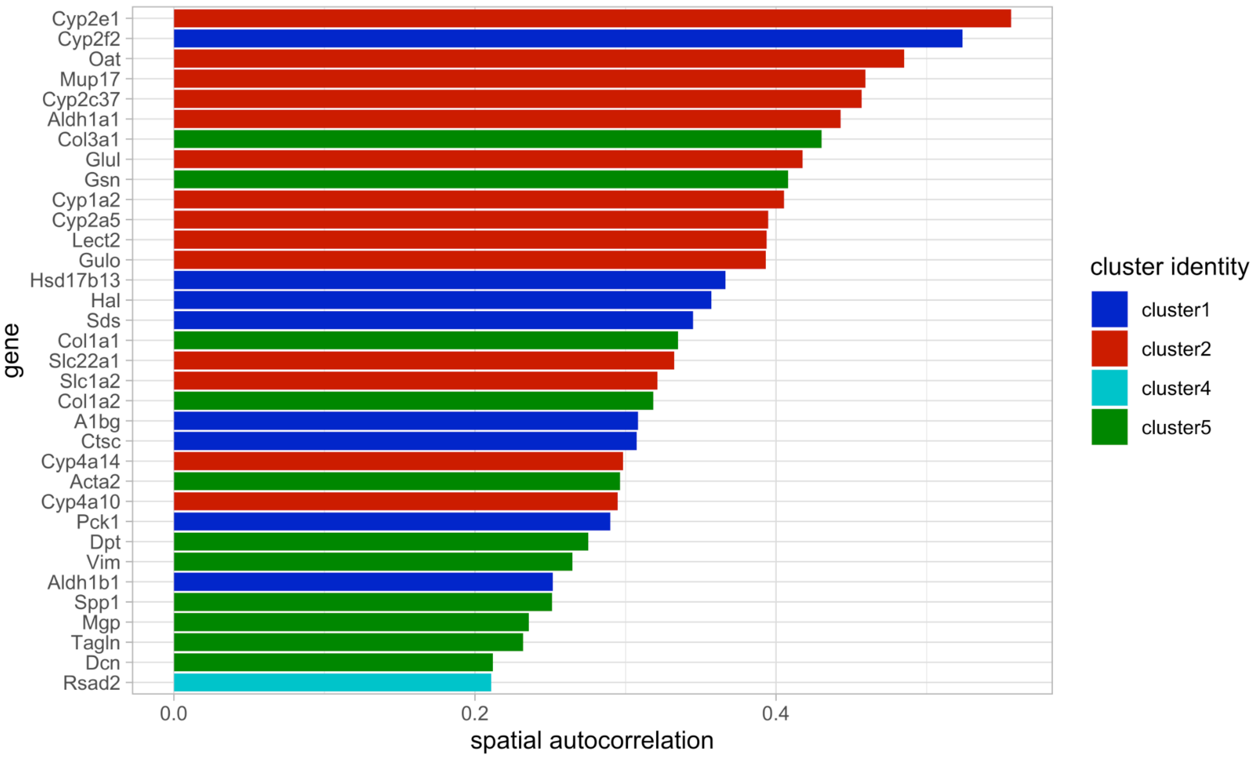

Supplementary Figure 11: a Heatmap depicting differentially expressed marker genes, associated with the GO-term "immune system process" (GO:0002376). Genes exhibiting highest elevation in either the portal area (cluster 1) or central area (cluster 2) are surrounded by a red box.These highlighted genes were selected to be analyzed with the bivariate expression by distance model. In **b** selected markers from **a** were subjected to bivariate expression analysis (methods). Numbers in curly brackets after the gene name indicate that 1: the full model does not perform significantly better than the reduced portal model, 2: the full model does not perform significantly better than the reduced central model, 3: the full model does not perform significantly better than either of the reduced models, 4: the full model does not outperform the most reduced model (intercept only), i.e., the gene expression can be taken as constant across the tissue w.r.t vein distances. Relative expression values for each gene are depicted in a color gradient ranging from low (dark) to high (light). The results from the likelihood-ratio tests (LRT) are presented in **Supplementary dataset 4**.

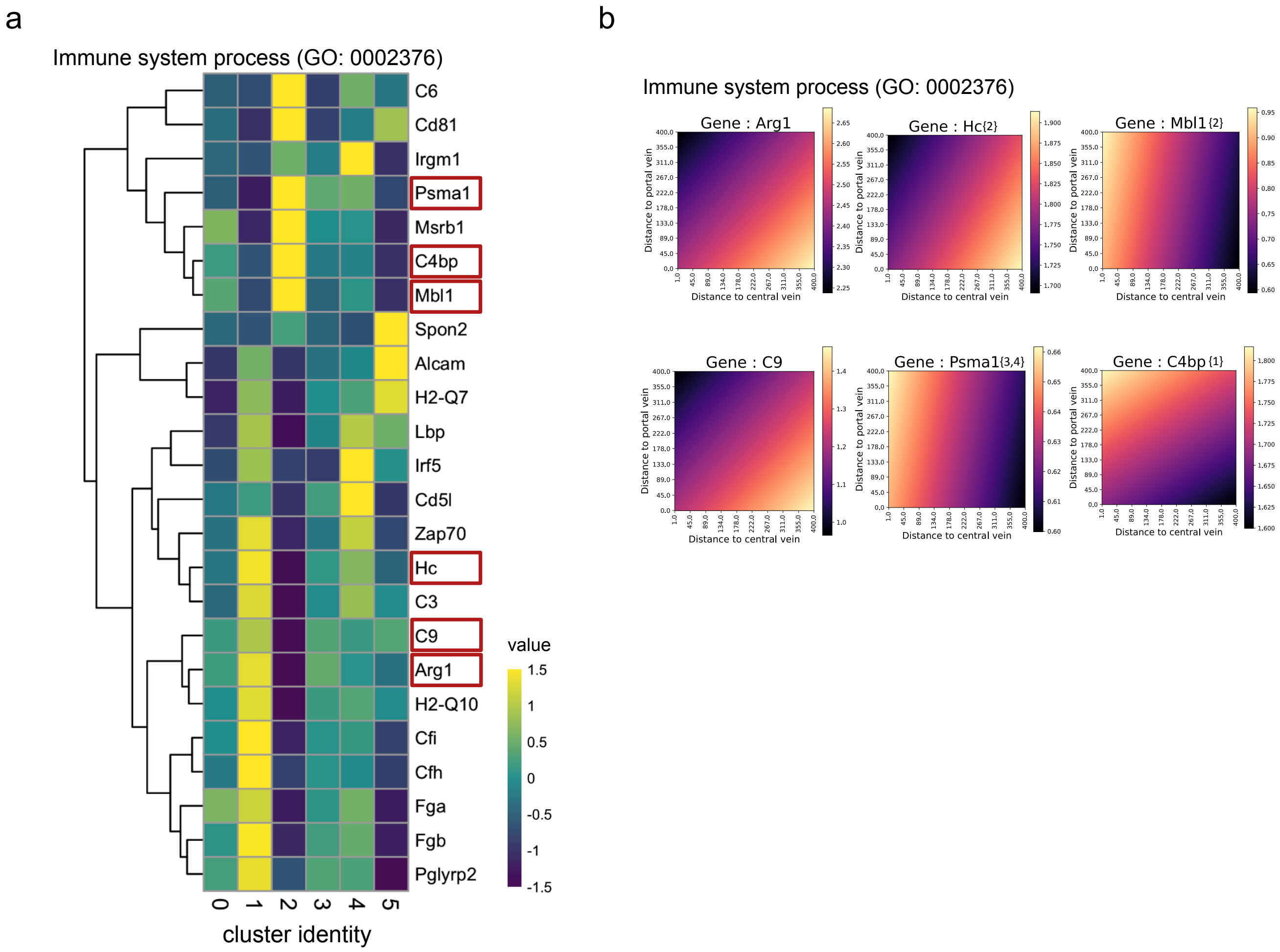

Supplementary figure 12: Visualization of marker genes of periportal annotated cluster 1. **a** *Sds* expression **b** *Cyp2f2* expression,and **c** module scores (see methods) for periportal markers of cluster 1 (bottom) on Hematoxylin- and Eosin-stained tissue for all sections of sample 1 to sample 3. Descending expression values/scores is visualized by decreasing opacity of spots and a color gradient from dark blue for high expression to white for low expression.

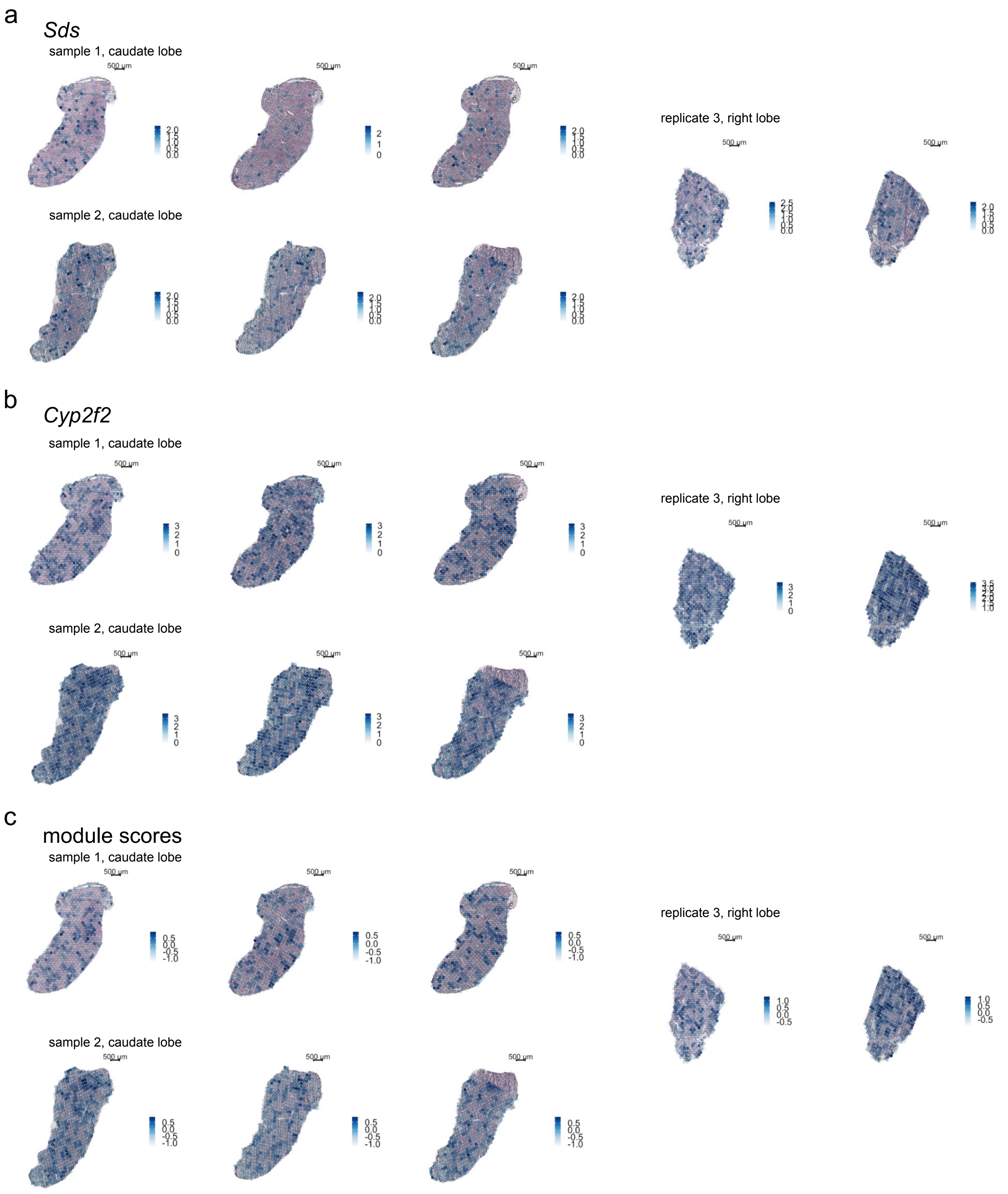

Supplementary figure 13: Visualization of marker genes of pericentral annotated cluster 2. **a** *Glul* expression **b** *Cyp2e1* expression,and **c** module scores (see methods) for pericentral markers of cluster 2 (bottom) on Hematoxylin- and Eosin-stained tissue for all sections of sample 1 to sample 3. Descending expression values/scores is visualized by decreasing opacity of spots and by a color gradient from dark red for high expression to white for low expression.

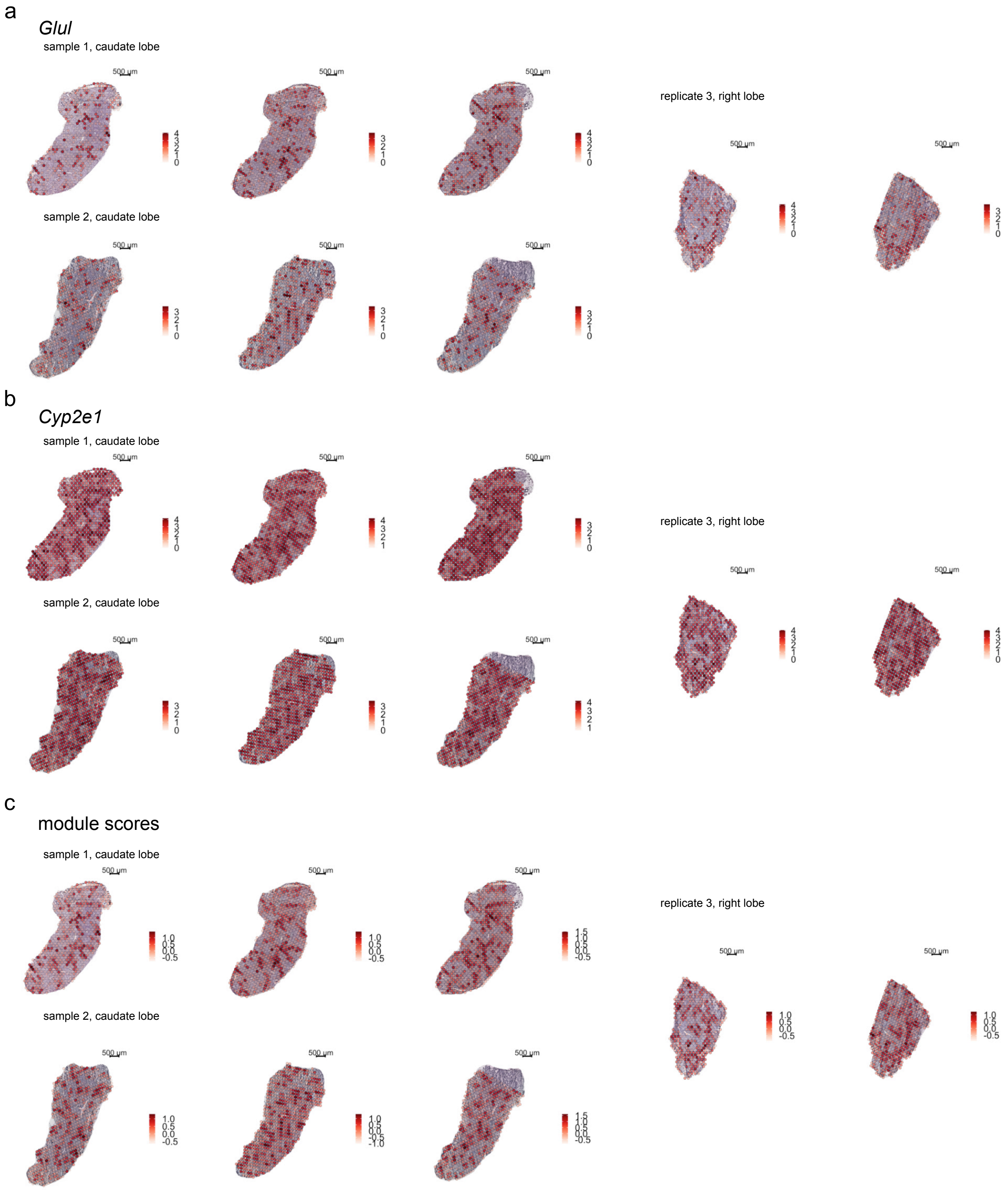

Supplementary Figure 14: Gene set enrichment of selected KEGG pathways for periportal and pericentral regions. Enrichment of established zonated metabolic pathways was determined and proportions of enriched genes sets were compared between the PP zone (cluster 1) and the PC zone (cluster 2) in our data. Negative values (red bars) represent enrichment in the central zone, while positive values (blue bars) represent enrichment in the portal area.

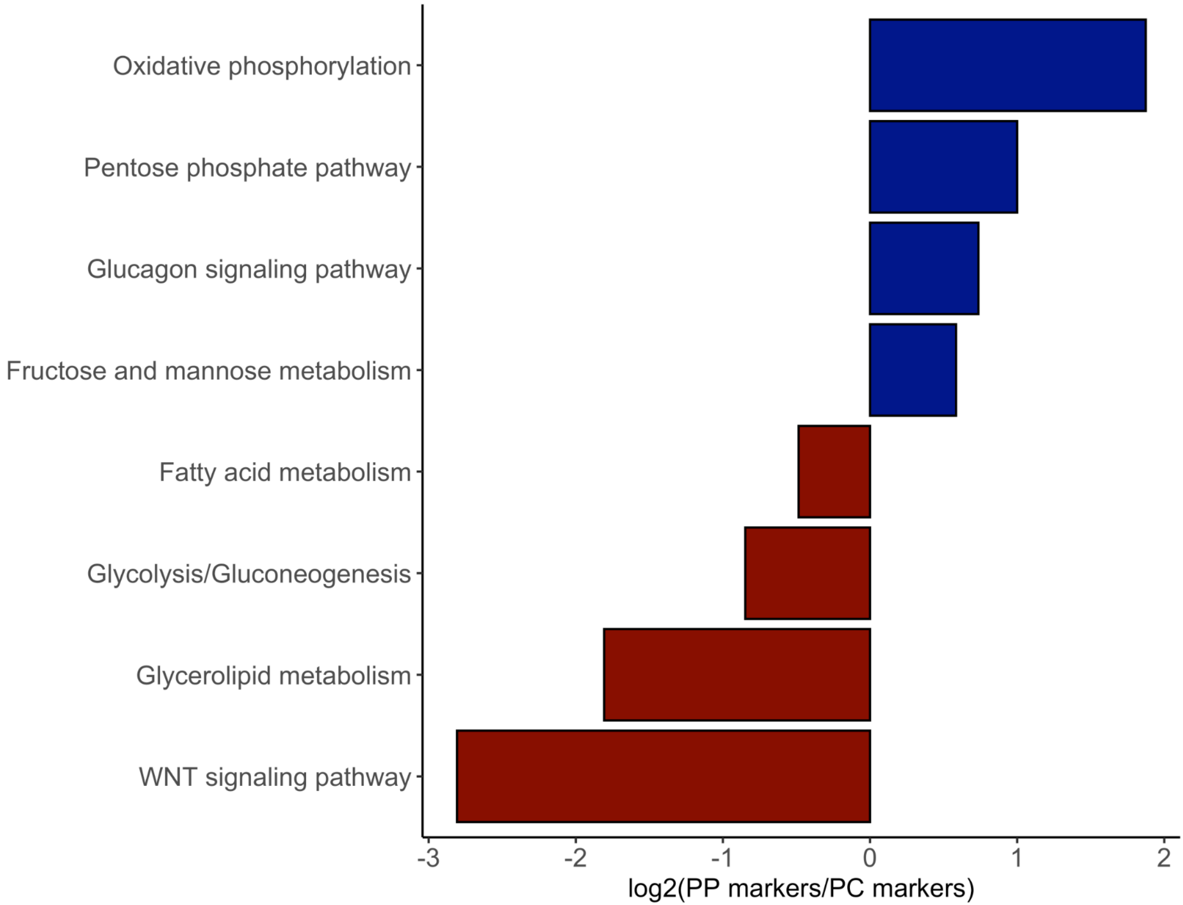

Supplementary figure 15: Shortlist of five marker genes of cluster 1 (portal vein) and cluster 2 (central vein) identified by unsupervised clustering of ST data. The central and portal markers depicted here, exhibit the highest logFC for the respective cluster in the ST data and were used in computational analyses of expression by distance plots and the computational annotation of vein types in the tissue.

central markers	portal markers
Glul	Sds
Oat	Cyp2f2
Slc1a2	Hal
Cyp2e1	Hsd17b13
Cyp2a5	Aldh1b1

Supplementary figure 16: Visualization of expression of pericentral marker genes identified by unsupervised clustering and DGEA of ST data across reconstructed spatial layers (1-9) of single cell data zonation matrix (Halpern et al., 2017). In combined plots, single cell expression data was scaled to the maximal expression value in the group of genes (see methods). Each pericentral marker gene is additionally plotted individually with original expression values of single cell data. Grey ribbons indicate standard error of gene expression across cells along layers.

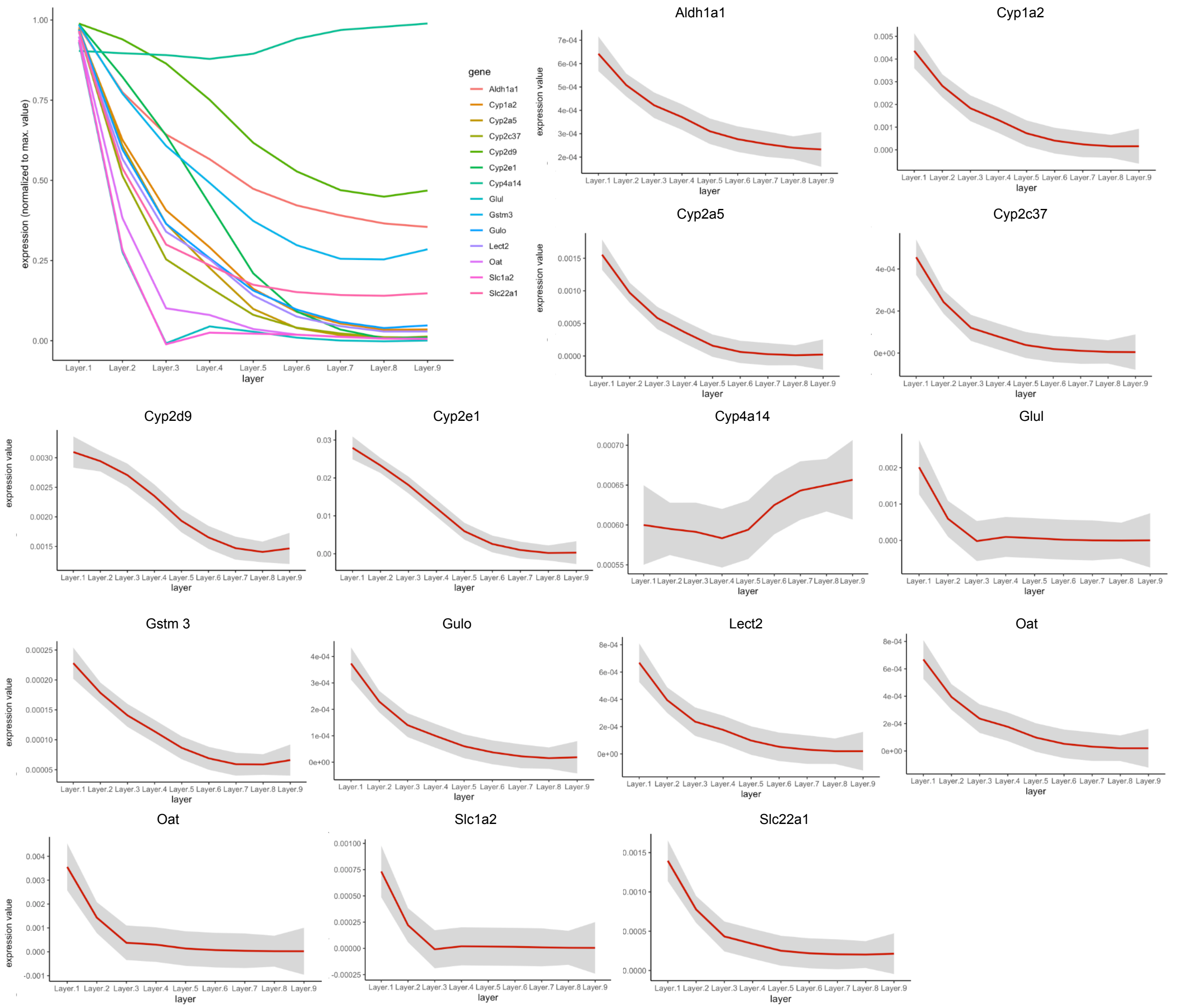

Supplementary figure 17: Visualization of expression of periportal marker genes identified by unsupervised clustering and DGEA of ST data across reconstructed spatial layers (1-9) of single cell data zonation matrix (Halpern et al., 2017). In combined plots, single cell expression data was scaled to the maximal expression value in the group of genes (see methods). Each periportal marker gene is additionally plotted individually with original expression values of single cell data. Grey ribbons indicate standard error of gene expression across cells along layers.

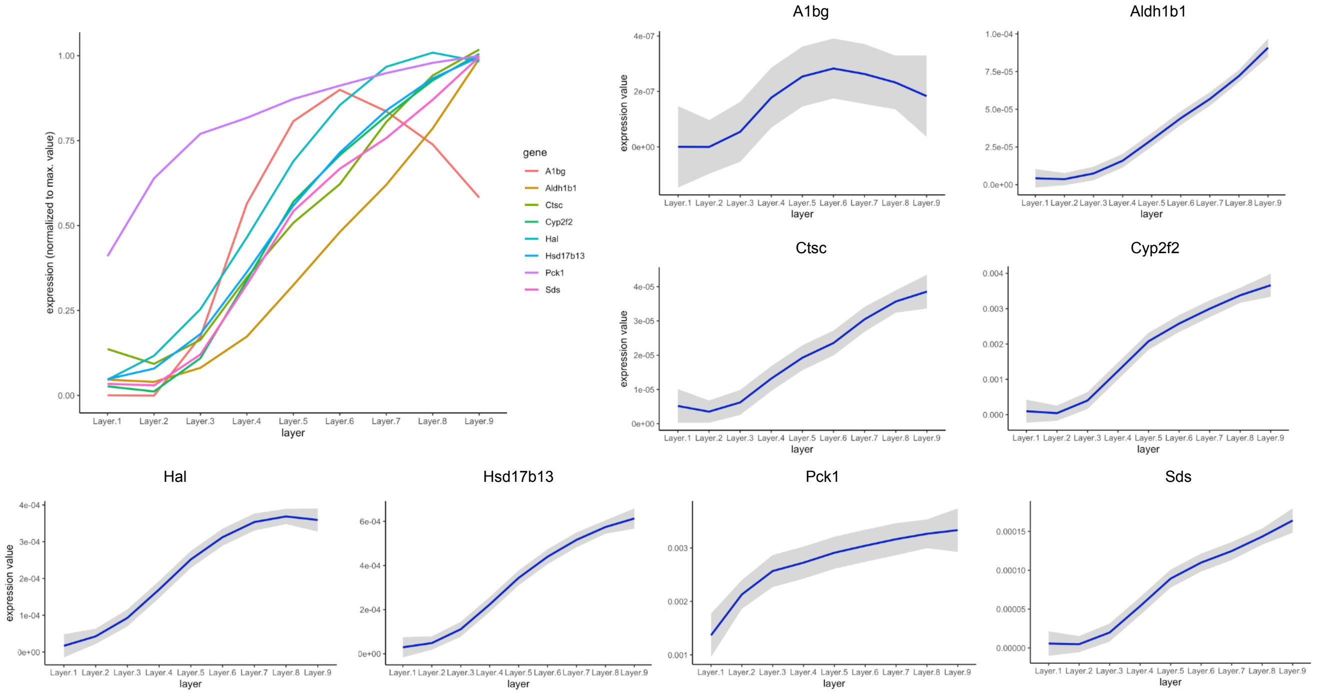

Supplementary figure 18: Expression by distance of annotated cell types of MCA single cell data from outer portal and central vein borders. Distances of pericentral hepatocytes from central (C) to portal (P) veins are depicted in the figure (on the left). Distances of periportal hepatocytes from portal (P)to central veins (C) are depicted in the figure (on the right). The standard error (SE) of spot proportion values along the distance to the vein is indicates by red (red) and blue (portal) ribbons.

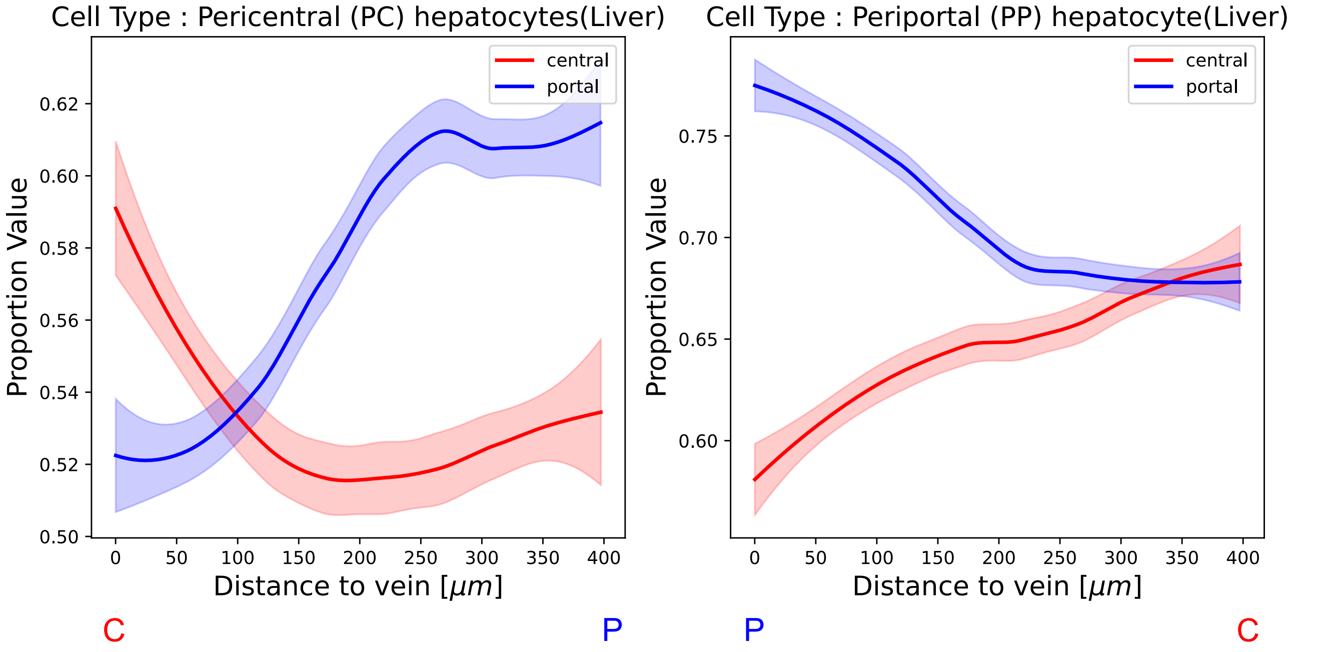

Supplementary figure 19: bivariate expression by distance analysis (methods) of selected genes. **a** glucagon target genes, **b** Wnt pathway target genes, **c** Ha-ras target genes, **d** chronic hypoxia target genes and **e** pituitary hormone target genes. For a detailed description of the plot interpretation see **Supplementary figure 11**. The results of likelihood-ratio tests (LRT) are presented in **Supplementary dataset 4**.

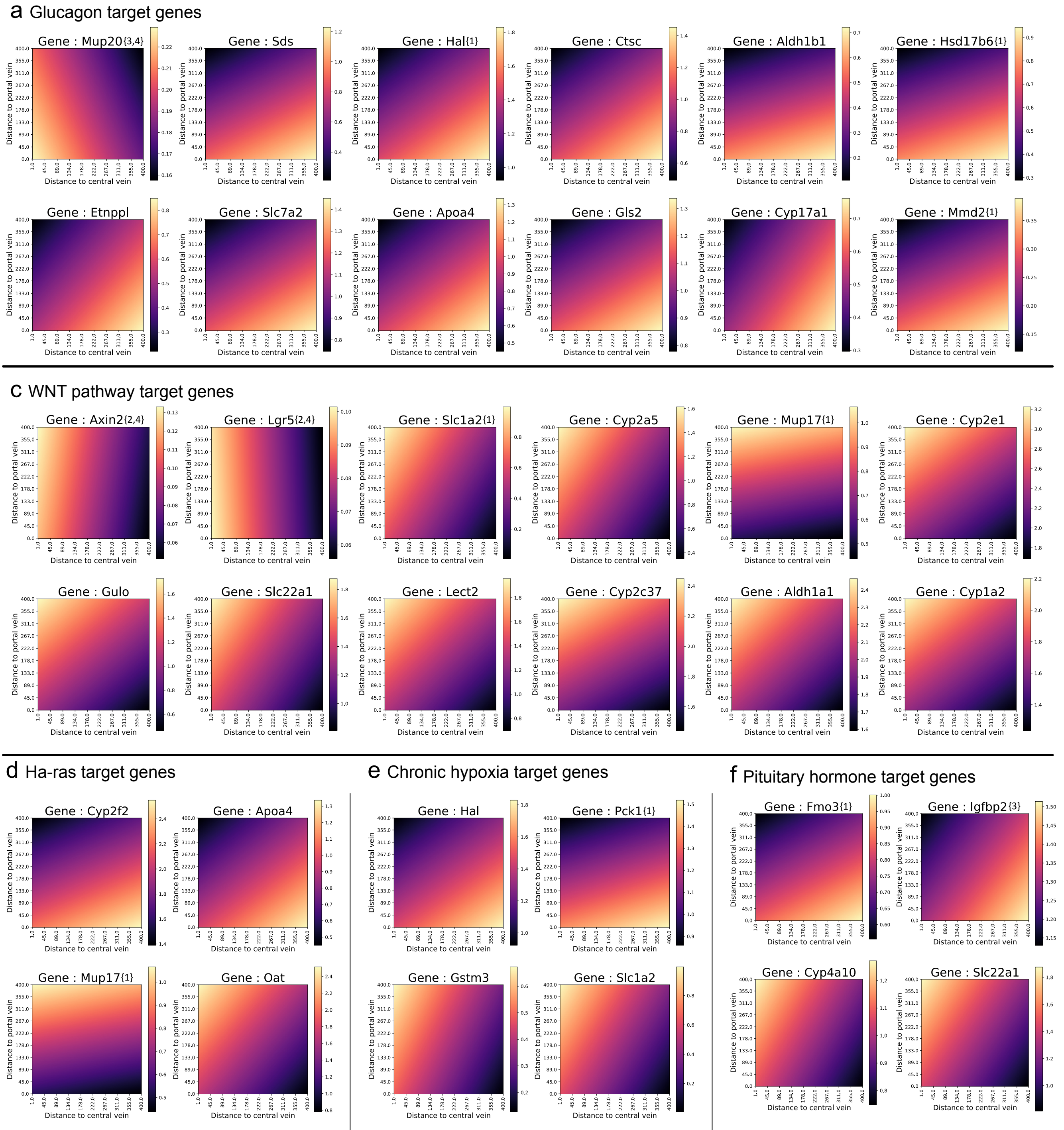

Supplementary figure 20: a ROC curve illustrating the performance of the expression-based vein type classifier. The blue line represents the average AUC taken over all the folds in the cross validation analysis. The red dashed line corresponds to the curve obtained from a completely random classifier. The gray shaded area represents the interval of the mean plus/minus one standard error. Av, AUC stands for average AUC and is the arithmetic mean taken across all folds.
b Barplots illustrating the results of the prediction accuracy of the cross validation analysis between sections (experiments). The blue color denotes correct annotations while grey signifies incorrect annotations. The red dotted line denotes 50% classification accuracy.
c Performance validation of binary vein classifier | For each sample prediction performance was evaluated by training according to an LOOCV. The performance is illustrated by the results of the accuracy of the cross validation analysis and area under the ROC curve (AUC).
A value of 1 denotes highest accuracy, while a value of 0 denotes lowest accuracy.

a

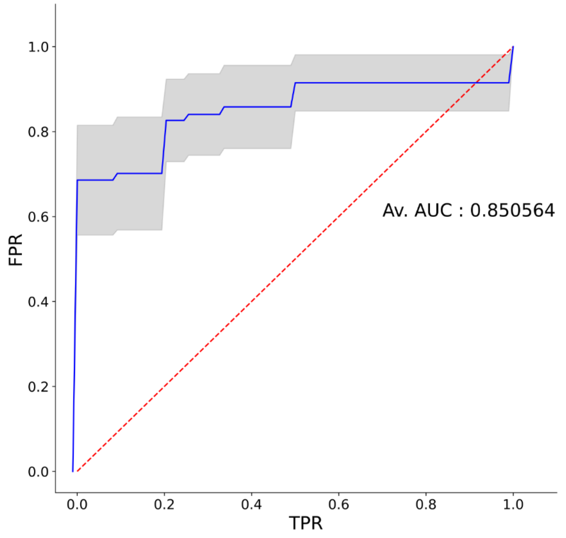

b

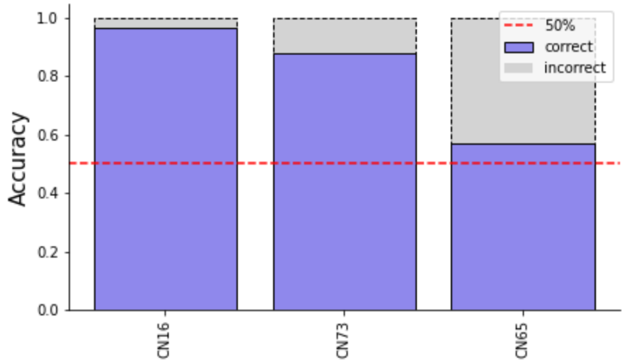

c

predict on	train on	accuracy	AUC
CN73-D1	CN65-E1, CN73-E2, CN65-D1, CN73-C1, CN16-D2, CN65-D2, CN16-E2	0.9333	0.9444
CN16-E2	CN65-E1, CN73-E2, CN65-D1, CN73-C1, CN16-D2, CN65-D2, CN73-D1	1	1
CN65-D2	CN65-E1, CN73-E2, CN65-D1, CN73-C1, CN16-D2, CN16-E2, CN73-D1	0.7143	0.8
CN16-D2	CN65-E1, CN73-E2, CN65-D1, CN73-C1, CN65-D2, CN16-E2, CN73-D1	0.9473	0.9886
CN73-C1	CN65-E1, CN73-E2, CN65-D1, CN16-D2, CN65-D2, CN16-E2, CN73-D1	0.9375	0.9524
CN65-D1	CN65-E1, CN73-E2, CN73-C1, CN16-D2, CN65-D2, CN16-E2, CN73-D1	1	1
CN73-E2	CN65-E1, CN65-D1, CN73-C1, CN16-D2, CN65-D2, CN16-E2, CN73-D1	0.8077	0.8333
CN65-E1	CN73-E2, CN65-D1, CN73-C1, CN16-D2, CN65-D2, CN16-E2, CN73-D1	0.2222	0.2857

Supplementary figure 21: Depiction of a selected region of orthogonal validation by immunological staining. **a** Shows the manual annotation of section 3, sample 3 (caudate lobe) (left), the computational prediction (middle, left) and an overlay of of the immunological staining for GS (middle, right) and SOX9 (right) on the computationally annotated section. Black scale bars indicate 500 µm. **b** shows immunological staining of a tissue section for DNA (Hoechst) (and central veins (GS), periportally located bile ducts (Sox9) and all images combined (merged). The white box highlights the excerpt shown in **supplementary figure 22** and white scale bars indicate 500 µm.

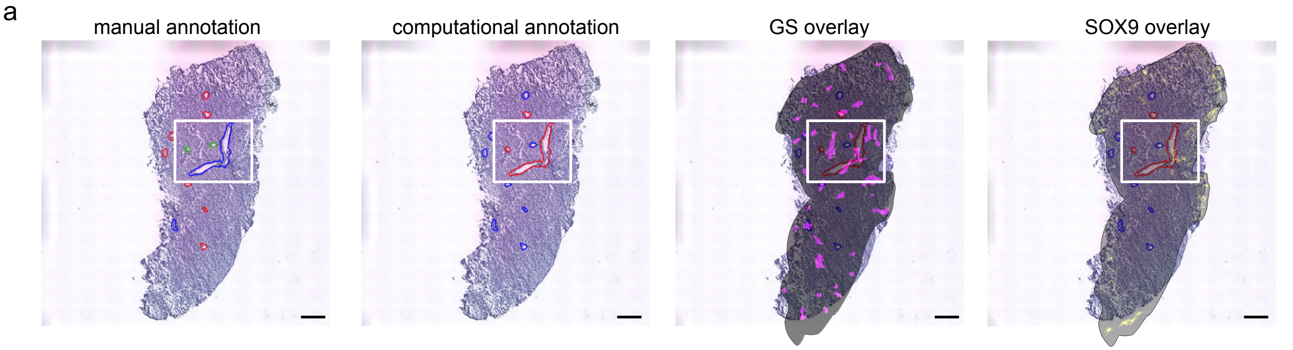

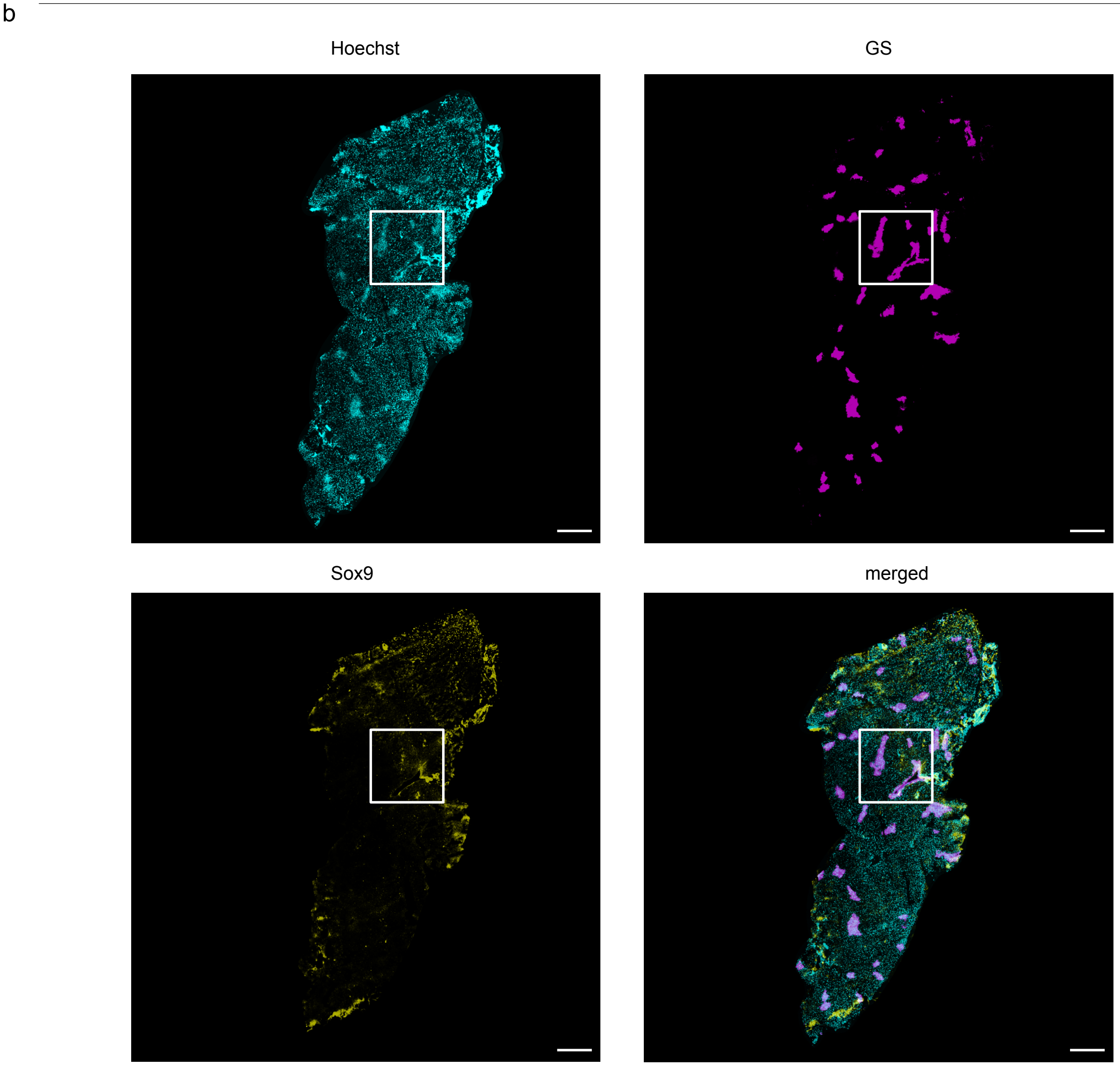

Supplementary figure 22: Orthogonal validation of selected central and portal veins. Depiction of a selected region of orthogonal validation by immunological staining. **a** Shows an overlay of the immunological staining for GS (left) and SOX9 (right) on the computationally annotated section 3 of sample 3 (caudate lobe). White scale bars indicate 100 µm. **b** shows the same structure in the immunologically stained images of one proximate section to sample 2 only, with the nuclear staining (left), the staining for central veins (GS, middle left), periportally located bile ducts (SOX9, middle right) and the overlay of all images (merged, right). White scale bars indicate 100 µm.

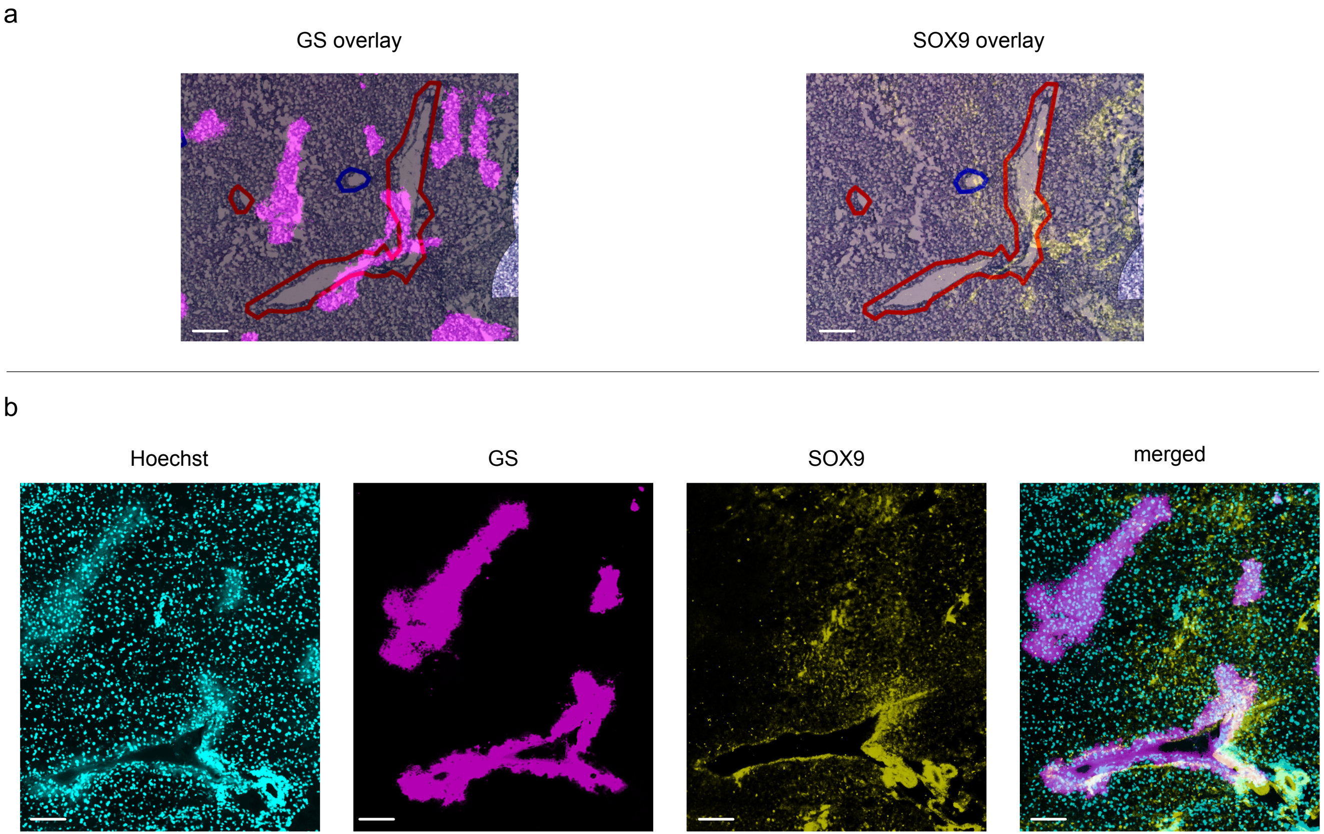

Supplementary figure 23: Visualization of spots under the tissue, assigned to cluster 5 by unsupervised clustering and visualization on all HE stained tissue sections. **a** Sample 1 with 3 sections of a part of a caudate lobe. **b** Sample 2 with 3 sections of a part of a caudate lobe and **c** sample 3 with 2 sections of a part of a right lobe.

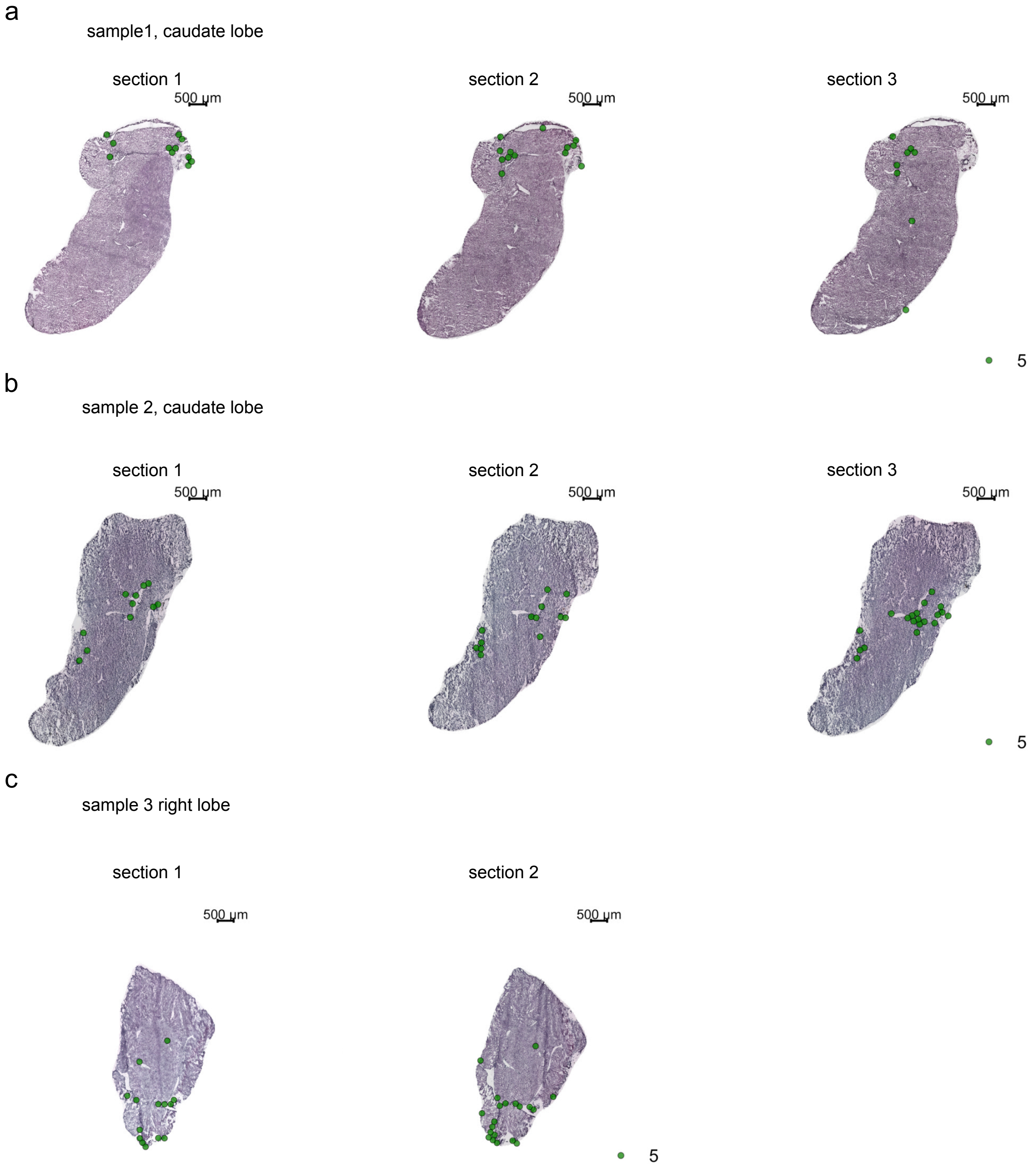

Supplementary figure 24: Visualization of four marker genes annotated to cluster 5 showing the highest positive logFC within the cluster: **a** *Vim,* **b** *Col3a1,* **c** *Col1a2* and **d** *Gsn* superimposed on all HE stained tissue sections of sample 1 to sample 3. Descending expression values/scores are visualized by decreasing opacity and a change from lighter to darker color.

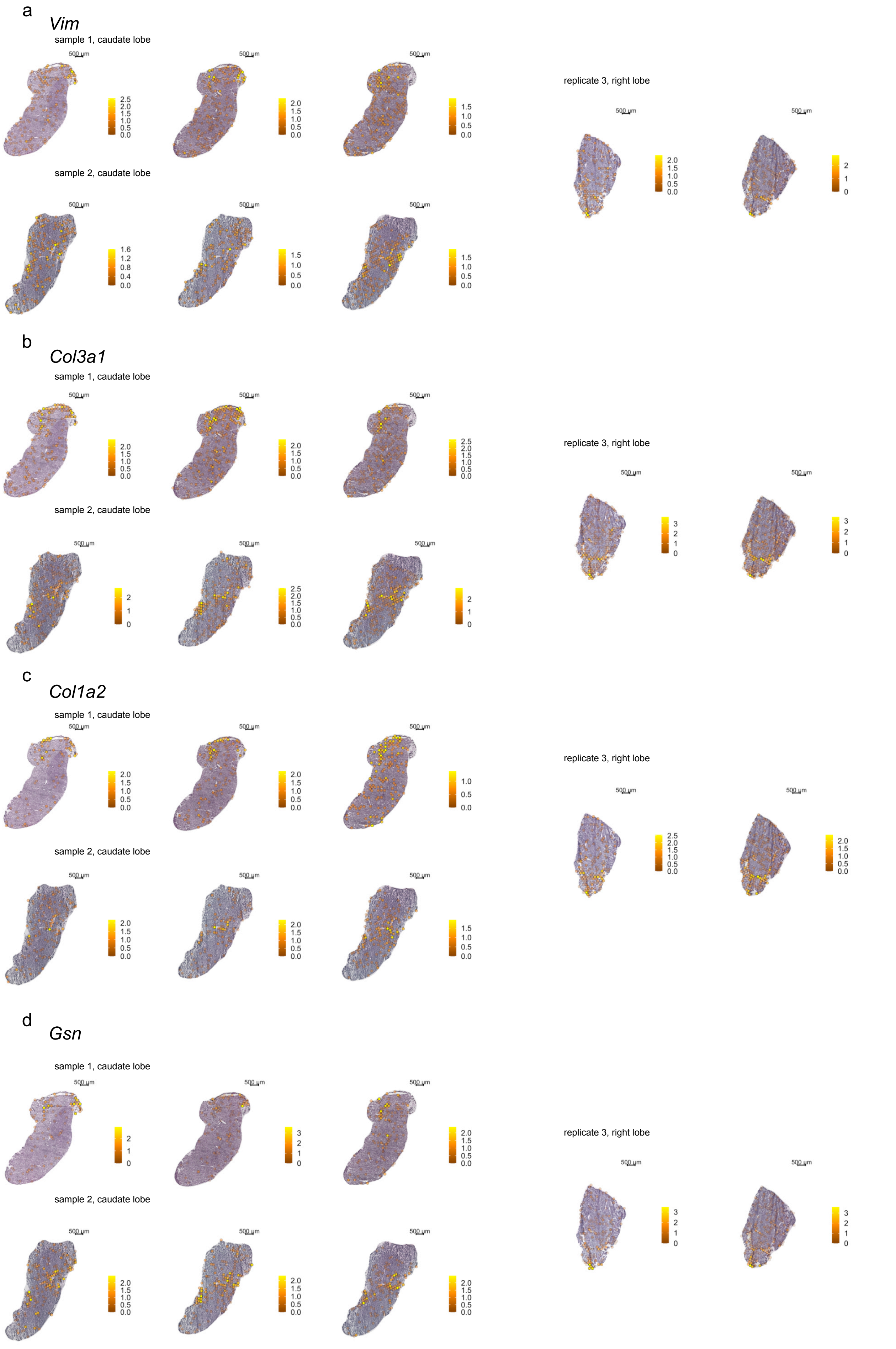

Supplementary figure 25: Feature-by-distance of genes correlated to distance to cluster 5
a feature-by-distance plot using cluster 5 as reference, here the top 15 genes with largest negative magnitude of the spearman correlation between expression and distance to cluster 5.
b feature-by-distance plot using cluster 5 as reference, here the top 15 genes with largest positive magnitude of the spearman correlation between expression and distance to cluster 5.

a

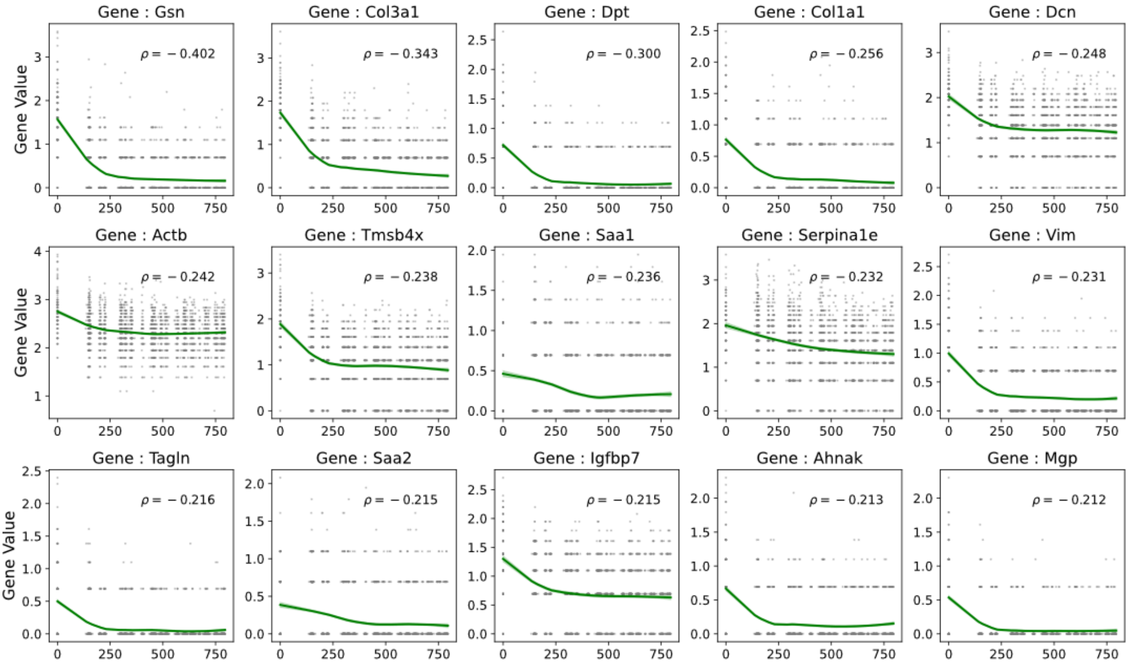

b

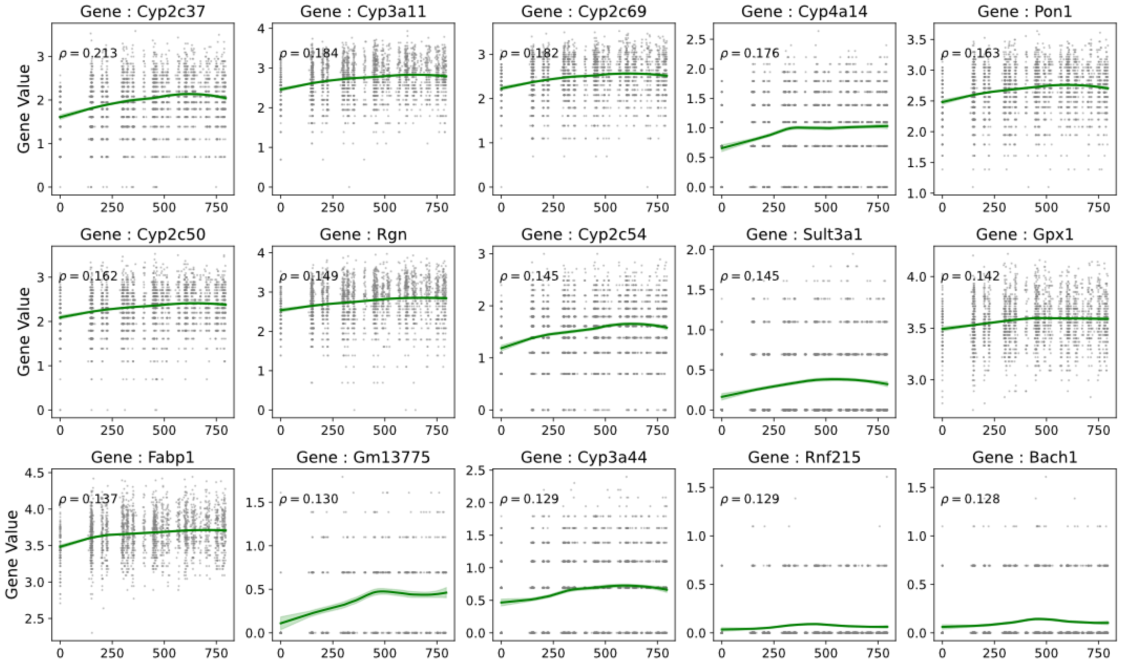

Supplementary figure 26: Visualization of pathway analysis on tissue sections. **a** Module scores for gene-sets with GO:0030199: "collagen fibril organization" annotation and **b** GO:0034097: "response to cytokine" (bottom), superimposed on all tissue sections and samples. Descending expression values/scores are in addition to change color visualized by decreasing opacity of spots.

a

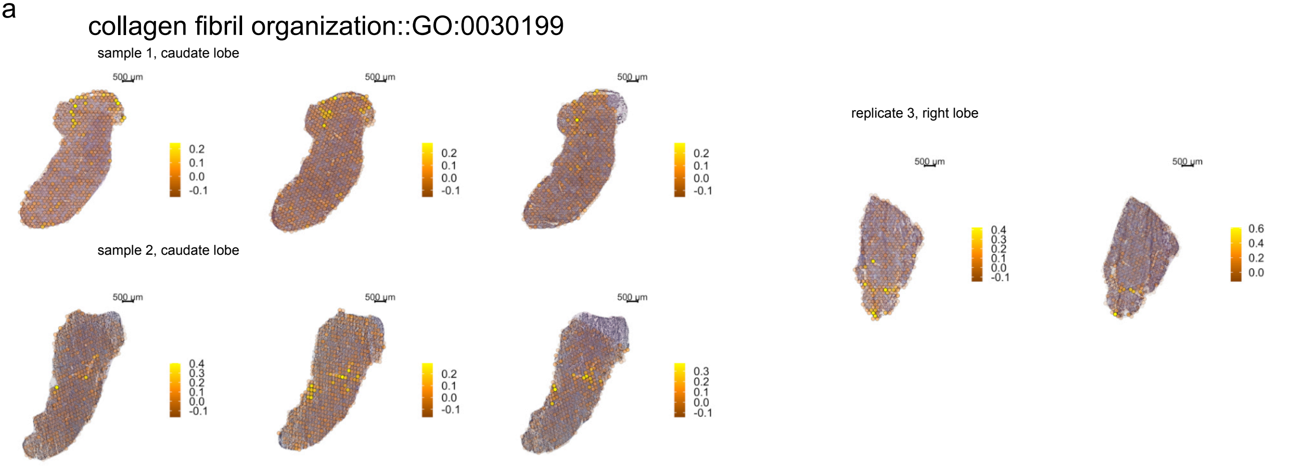

b

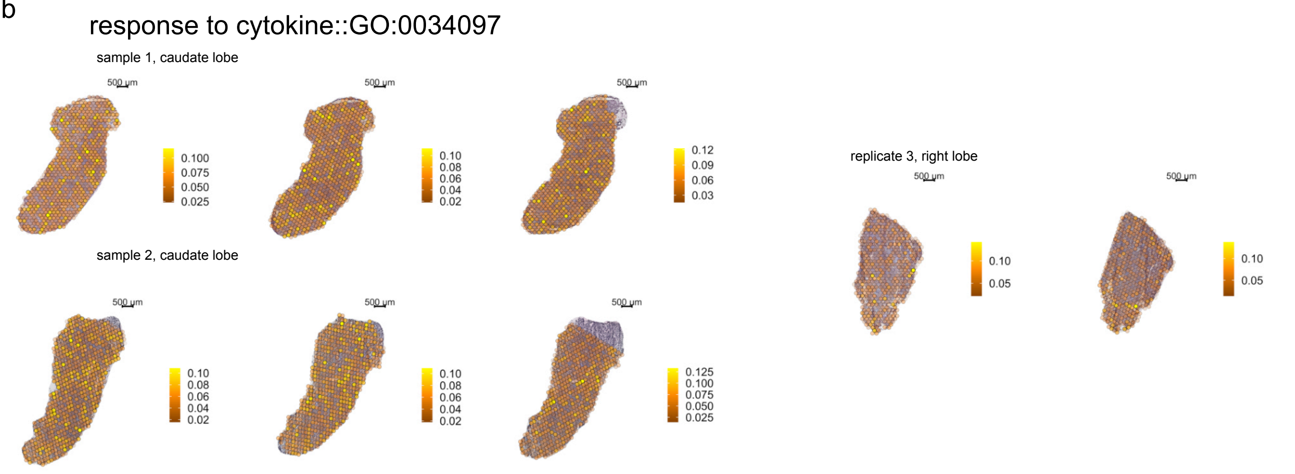

Supplementary figure 27: Intersection of marker genes of cluster 5 and annotated NPC types and mesenchymal cell types. The list of cluster 5 markers from the ST data (yellow) was compared to markers of annotated cell types from [PMID: 30222169] in blue and [PMID: 31722201] in green. Shared markers between cluster 5 and cell types of [PMID: 30222169] and [PMID:31722201] are marked with a cross in the respective cell.

	PMID: 30222169							PMID: 31722201		
Gene	Endothelial cells	Tcells	Kupffercells	Bcells	LCMs	pDCs	Neutrophils	HSCs	Fibroblasts	VSMCs
Gsn						X			X	
Dpt									X	
Mgp									X	
Col1a1									X	
Tagln										X
Col3a1									X	
Vim									X	
Col1a2									X	
H2-Eb1				X	X					
Crip1					X					X
Acta 2										X
Ahnak									X	
Tmsb4x					X					
Timp2									X	
Dcn								X		
H2-Aa				X	X					
Lum									X	
H2-Ab1				X	X					
Cd74				X	X					
Igfbp7	X							X		
Sparc	X									
Bgn	X							X		
Col14a1								X		
Spp1										
Timp3	X								X	X
Txnip									X	

Supplementary table 1: Spearman correlation values of module score values of periportal and pericentral gene markers from single cell data (hep.pp_MCA, hep.pc_MCA) and module score values of pericentral and periportal markers from spatial transcriptomics data (cluster1_ST, cluster 2_ST) using spatial transcriptomics data.

	cluster1_ST	hep.pp_MCA	cluster2_ST	hep.pc_MCA
**cluster1_ST**	1	0.237944152960821	-0.747074463978828	-0.150505061278013
**hep.pp_MCA**	0.237944152960821	1	-0.0108272714188795	0.44290617681838
**cluster2_ST**	-0.747074463978828	-0.0108272714188795	1	0.150868310269106
**hep.pc_MCA**	-0.150505061278013	0.44290617681838	0.150868310269106	1

Supplementary table 2: Spearman correlation values of module score values of periportal and pericentral gene markers from single cell data (hep.pp_MCA, hep.pc_MCA) and module score values of pericentral and periportal markers from spatial transcriptomics data (cluster1_ST, cluster 2_ST) using mouse cell atlas (MCA) data.

	cluster1_ST	hep.pp_MCA	cluster2_ST	hep.pc_MCA
**cluster1_ST**	1	0.211384034296339	0.0991093609396526	0.0515614925278205
**hep.pp_MCA**	0.211384034296339	1	0.245429161314913	0.674256868173379
**cluster2_ST**	0.0991093609396526	0.245429161314913	1	0.110786078630394
**hep.pc_MCA**	0.0515614925278205	0.674256868173379	0.110786078630394	1
