# Supplementary material for: Spatial Transcriptomics to define transcriptional patterns of zonation and structural components in the mouse liver
Source: Nat Commun. 2021 Dec 2;12:7046. doi: 10.1038/s41467-021-27354-w (PMC8640072; doi:10.1038/s41467-021-27354-w)
Supplement: Supplementary file 3 — Description of Additional Supplementary File [file 41467_2021_27354_MOESM3_ESM.pdf]

Filename: Supplementary dataset 1
Description:
Results of Differential Gene Expression Analysis (DGEA) between spatial clusters, identified by unsupervised clustering of ST data. Differential gene expression was performed between clusters of spatial data using a Wilcoxon Rank sum test. Only genes with an average log foldchange of 0.5 were tested. Only genes with an adjusted p-values < 0.05 are shown. P-value correction was performed using a Bonferroni correction based on the total number of genes in the dataset.

Filename: Supplementary dataset 2
Description:
Correlation matrix of genes of all identified clusters (0-5) of spatial data using Pearson correlation. Positive values indicate positive correlation of expression (spatial co-localization), while negative values indicate negative correlation of expression (spatial segregation).

Filename: Supplementary dataset 3
Description:
Results of Spatial autocorrelation of genes (see methods). A threshold of 0.2 was set to consider spatial autocorrelation of genes of all identified clusters (0-5) of spatial data using Pearson correlation. The cluster ID indicates which cluster shows differential expression of each respective gene.

Filename: Supplementary dataset 4
Description:
Results of likelihood ratio of test for model performance comparisons of the univariate and bivariate expression by distance models as described in the corresponding methods section in the manuscript. One degree of freedom was used for each of the reduced models (univariate model) and two degrees of freedom were used for the full model (bivariate model). The outcome of the likelihood ratio test (presented as a p-value), states whether the full model significantly (p-value < 0.05) outperforms the reduced model, accounting for the additional model parameters. This was performed for marker genes of zonation (cluster 1 and cluster 2), Glucagon, WNT, Ha-ras hypoxia and pituitary hormone target genes as well as immune system process related genes (GO:0002376).

Filename: Supplementary dataset 5
Description:
Estimated probabilities for central and portal vein annotations for all veins across samples and sections. Each vein number refers to a visually annotated vein in the tissue and can be reproduced using the hepaquery package.

Filename: Supplementary dataset 6
Description:
Results of GO-term analysis as for enriched marker genes of cluster 5 using the g:Profiler package, described in the methods section of the manuscript. Significance was adjusted using g:SCS (Set Counts and Sizes), as originally described by the authors of the g:Profiler package.

Filename: Supplementary dataset 7
Description:
List of 5000 most variable genes of single cell data set used for stereoscope integration as described in the methods section of the manuscript.
