# Supplementary material for: Spatial Transcriptomics to define transcriptional patterns of zonation and structural components in the mouse liver
Source: Nat Commun. 2021 Dec 2;12:7046. doi: 10.1038/s41467-021-27354-w (PMC8640072; doi:10.1038/s41467-021-27354-w)
Supplement: Supplementary file 11 — Reporting summary [file 41467_2021_27354_MOESM11_ESM.pdf]

nature research

Corresponding author(s): Johan Ankarklev, Franziska Hildebrandt

Last updated by author(s): 10/29/2021

Reporting Summary

Nature Research wishes to improve the reproducibility of the work that we publish. This form provides structure for consistency and transparency in reporting. For further information on Nature Research policies, see our Editorial Policies and the Editorial Policy Checklist.

Statistics

For all statistical analyses, confirm that the following items are present in the figure legend, table legend, main text, or Methods section.

n/a	Confirmed	
[ ]	[x]	The exact sample size (*n*) for each experimental group/condition, given as a discrete number and unit of measurement
[ ]	[x]	A statement on whether measurements were taken from distinct samples or whether the same sample was measured repeatedly
[ ]	[x]	The statistical test(s) used AND whether they are one- or two-sided<br>*Only common tests should be described solely by name; describe more complex techniques in the Methods section.*
[x]	[ ]	A description of all covariates tested
[ ]	[x]	A description of any assumptions or corrections, such as tests of normality and adjustment for multiple comparisons
[ ]	[x]	A full description of the statistical parameters including central tendency (e.g. means) or other basic estimates (e.g. regression coefficient) AND variation (e.g. standard deviation) or associated estimates of uncertainty (e.g. confidence intervals)
[ ]	[x]	For null hypothesis testing, the test statistic (e.g. *F*, *t*, *r*) with confidence intervals, effect sizes, degrees of freedom and *P* value noted<br>*Give P values as exact values whenever suitable.*
[x]	[ ]	For Bayesian analysis, information on the choice of priors and Markov chain Monte Carlo settings
[ ]	[x]	For hierarchical and complex designs, identification of the appropriate level for tests and full reporting of outcomes
[ ]	[x]	Estimates of effect sizes (e.g. Cohen's *d*, Pearson's *r*), indicating how they were calculated

Our web collection on statistics for biologists contains articles on many of the points above.

Software and code

Policy information about availability of computer code

Data collection	raw data was processed using the open source ST Pipeline (v 1.7.6) and STAR (v 2.6.1e)
Data analysis	Data was analyzed using the following software and packages: R (v 4.0.2), STUtility (v 0.1.0), Seurat (v 3.2.2), corrplot (v 0.84), gprofiler2 (v 0.1.0), ggplot2 (v 3.3.2), stereoscope (v.0.3 (commit: aacd5f775b73b138e504c35ff0cb3ffafbfc78ff), python (v 3.8.3), hepaquery (v 0.0.1), scikit-misc package (v 0.1.3), statsmodels package (v 0.11.1), louvain algorithm, loess algorithm

For manuscripts utilizing custom algorithms or software that are central to the research but not yet described in published literature, software must be made available to editors and reviewers. We strongly encourage code deposition in a community repository (e.g. GitHub). See the Nature Research guidelines for submitting code & software for further information.

Data

Policy information about availability of data

All manuscripts must include a data availability statement. This statement should provide the following information, where applicable:

- Accession codes, unique identifiers, or web links for publicly available datasets
- A list of figures that have associated raw data
- A description of any restrictions on data availability

The datasets generated during and/or analyzed during the current study are available in the doi-minting zenodo repository "Spatial Transcriptomics to define transcriptional patterns of zonation and structural components in the liver" [https://zenodo.org/record/5045689] 72. The raw expression data and spot files can be accessed at the Gene Expression Omnibus database with the accession code GSE165141 [https://www.ncbi.nlm.nih.gov/geo/query/acc.cgi?acc=GSE165141]. The data used for comparative analysis of previously published data can be accessed at 12 and Gene Expression omnibus (accession code GSE84498 [https://www.ncbi.nlm.nih.gov/geo/query/acc.cgi?acc=GSE84498]) as well as at 41,[http://bis.zju.edu.cn/MCA/] with raw data accessible at Gene Expression omnibus (accession code GSE108097 [https://www.ncbi.nlm.nih.gov/geo/query/acc.cgi?acc=GSE108097]) and 29 with raw data accessible at Gene Expression omnibus

(accession code GSE137720 [https://www.ncbi.nlm.nih.gov/geo/query/acc.cgi?acc=GSE137720]). Where applicable, gene ontology terms (GO) were extracted from the gene ontology browser of the Mouse Genome Informatics (MGI) database [http://www.informatics.jax.org/]. Source data are provided with this paper

Field-specific reporting

Please select the one below that is the best fit for your research. If you are not sure, read the appropriate sections before making your selection.

[x] Life sciences   [ ] Behavioural & social sciences   [ ] Ecological, evolutionary & environmental sciences

For a reference copy of the document with all sections, see nature.com/documents/nr-reporting-summary-flat.pdf

Life sciences study design

All studies must disclose on these points even when the disclosure is negative.

Sample size	Samples were chosen according to the number of the availability of experiments. Each Spatial Transcriptomics slide contains 6 sub-arrays. All samples resulting in cDNA libraries of correct size and concentration of all performed experiments were considered. Since the sequencing data resulted in satisfactory depth for two liver replicates of mouse liver tissue we argued the produced data is sufficient to support the conclusions made in the study.
Data exclusions	Only data that was handled the same way during samples freezing and library preparation was considered for consistent final analysis. Therefore, samples "CN16-D1" and "CN16-E1" were excluded from the final analysis due to a different freezing protocol for the liver sample preparation. Samples "CN65-C1" and "CN65-C2" were excluded from the data, since they followed a different protocol before Hematoxylin and Eosin staining during optimization processes. No other data was excluded from the study.
Replication	To control for biological variation and increase reproducibility, liver samples from two individual mice and lobes were used during this study. All attempts at replication were successful.
Randomization	Randomization was not applicable to this study, since it does not include clinical samples or comparative hypotheses between different groups.
Blinding	Blinding was not applicable to this study, since grouping of data did not take place.

Reporting for specific materials, systems and methods

We require information from authors about some types of materials, experimental systems and methods used in many studies. Here, indicate whether each material, system or method listed is relevant to your study. If you are not sure if a list item applies to your research, read the appropriate section before selecting a response.

Materials & experimental systems

n/a	Involved in the study	
[ ]	[x]	Antibodies
[x]	[ ]	Eukaryotic cell lines
[x]	[ ]	Palaeontology and archaeology
[ ]	[x]	Animals and other organisms
[x]	[ ]	Human research participants
[x]	[ ]	Clinical data
[x]	[ ]	Dual use research of concern

Methods

n/a	Involved in the study	
[x]	[ ]	ChIP-seq
[x]	[ ]	Flow cytometry
[x]	[ ]	MRI-based neuroimaging

Antibodies

Antibodies used	Primary antibodies:<br>1. GS (1:1000; ab73593, Abcam)<br>2. SOX9 (1/100; AB5535, Millipore)<br>3. F4/80 (1:100; MCA497RT, BioRad)<br>4. HNF4α (1/100; sc6556, Santa Cruz)<br>5. CD31 (1:200; MEC13.3, BioLegend)<br>Secondary antibodies:<br>6. anti-rabbit AlexaFluor 488<br>7. anti-rabbit/anti-goat/anti-rat AlexaFluor 647 (1:1000, Invitrogen)
Validation	1. GS: validated in target organism (mus musculus), information from the vendor (64 citations): https://www.citeab.com/antibodies/733545-ab73593-anti-glutamine-synthetase-antibody, exemplary publiction: DOI: 10.1016/j.immuni.2020.08.004<br>2. SOX9: validated in target organism (mus musculus), information from the vendor (44 citations): https://www.sigmaaldrich.com/SE/en/search/hpa001758?focus=papers&page=1&perPage=30&sort=relevance&term=HPA001758&type=citation_search, exemplary

publication: DOI: 10.1152/ajplung.00144.2014
3. F4/80: validated in target organism (mus musculus), information from the vendor (121 citations): https://www.bio-rad-antibodies.com/monoclonal/mouse-f4-80-antibody-cl-a3-1-mca497.html?f=purified#references, exemplary publication: DOI: 10.1002/eji.1830111013
4. HNF4α: validated in target organism (mus musculus), information from the vendor (95 citations): https://www.scbt.com/p/hnf-4alpha-antibody-c-19, exemplary publication: DOI: 10.1371/journal.pone.0140020
5. CD31: validated in target organism (mus musculus), information from the vendor (40 citations): https://www.biolegend.com/en-us/products/alexa-fluor-647-anti-mouse-cd31-antibody-3094, exemplary publication: DOI: 10.1038/s41598-018-19248-7

Animals and other organisms

Policy information about studies involving animals; ARRIVE guidelines recommended for reporting animal research

Laboratory animals	The study involved female Mus musculus species, strain C57BL/6, euthanized between 8 and 12 weeks of age.
Wild animals	The study did not involve wild animals
Field-collected samples	The study did not involve samples collected from the field
Ethics oversight	The Regional Animal Research Ethical Board, Stockholm, Sweden, approved experimental procedures and protocols involving extraction of organs from mice (N135/15, N78/16 and 9707-2018), following proceedings described in EU legislation (Council Directive 2010/63/EU). The mice used in this study were kept at a 12h night/day cycle and 22°C ambient temperature, with free access to food and water under specific pathogen-free conditions at the Experimental Core Facility, Stockholm University and were euthanized between 8 and 12 weeks of age.

Note that full information on the approval of the study protocol must also be provided in the manuscript.
